# Cattle genome-wide analysis reveals genetic signatures in trypanotolerant N’Dama

**DOI:** 10.1186/s12864-017-3742-2

**Published:** 2017-05-12

**Authors:** Soo-Jin Kim, Sojeong Ka, Jung-Woo Ha, Jaemin Kim, DongAhn Yoo, Kwondo Kim, Hak-Kyo Lee, Dajeong Lim, Seoae Cho, Olivier Hanotte, Okeyo Ally Mwai, Tadelle Dessie, Stephen Kemp, Sung Jong Oh, Heebal Kim

**Affiliations:** 1 Department of Agricultural Biotechnology and Research Institute of Agriculture and Life Sciences, Seoul National University, Seoul, 08826 Republic of Korea; 2 C&K Genomics, Seoul National University Research Park, Seoul, 151-919 Republic of Korea; 3 Clova, NAVER Corp., Seongnam, 13561 Republic of Korea; 4 Interdisciplinary Program in Bioinformatics, Seoul National University, Seoul, 08826 Republic of Korea; 5 Department of Animal Biotechnology, Chonbuk National University, Jeonju, 66414 Republic of Korea; 6 Division of Animal Genomics and Bioinformatics, National Institute of Animal Science, RDA, Jeonju, 55365 Republic of Korea; 7 University of Nottingham, School of Life Sciences, Nottingham, NG7 2RD UK; 8 International Livestock Research Institute, Addis Ababa, Ethiopia; 9 International Livestock Research Institute, Box 30709-00100, Nairobi, Kenya; 10 The Centre for Tropical Livestock Genetics and Health, The Roslin Institute, University of Edinburgh, Easter Bush Campus, Edinburgh, Scotland UK; 11 National Institute of Animal Science, RDA, Wanju, 55365 Republic of Korea

**Keywords:** Cattle genome, Trypanotolerant N’Dama, SNPs, Genetic signatures, Comparative genome-wide analysis

## Abstract

**Background:**

Indigenous cattle in Africa have adapted to various local environments to acquire superior phenotypes that enhance their survival under harsh conditions. While many studies investigated the adaptation of overall African cattle, genetic characteristics of each breed have been poorly studied.

**Results:**

We performed the comparative genome-wide analysis to assess evidence for subspeciation within species at the genetic level in trypanotolerant N’Dama cattle. We analysed genetic variation patterns in N’Dama from the genomes of 101 cattle breeds including 48 samples of five indigenous African cattle breeds and 53 samples of various commercial breeds. Analysis of SNP variances between cattle breeds using wMI, XP-CLR, and XP-EHH detected genes containing N’Dama-specific genetic variants and their potential associations. Functional annotation analysis revealed that these genes are associated with ossification, neurological and immune system. Particularly, the genes involved in bone formation indicate that local adaptation of N’Dama may engage in skeletal growth as well as immune systems.

**Conclusions:**

Our results imply that N’Dama might have acquired distinct genotypes associated with growth and regulation of regional diseases including trypanosomiasis. Moreover, this study offers significant insights into identifying genetic signatures for natural and artificial selection of diverse African cattle breeds.

**Electronic supplementary material:**

The online version of this article (doi:10.1186/s12864-017-3742-2) contains supplementary material, which is available to authorized users.

## Background

Cattle are vital resources for African economy and society. Approximately 150 breeds of indigenous cattle have been found in sub-Saharan Africa [1]. Indigenous African cattle which have inhabited geographically isolated region for a long time have been subjected to the environmental pressure. This imposed strong adaptive constraints to African cattle, and thus led to selection of the fitter individuals to the harsh conditions [2]. In particular, some breeds (e.g. Gobra zebu and N’Dama) have acquired tolerance to local diseases that is known to significantly decrease the survival and productivity of African livestock [3]. In addition to the environmental factors, artificial selection has resulted in characteristic phenotypes in a few breeds (e.g. Ankole, Boran, Kenana and Ogaden), which enhanced the production of dairy products and beef [4, 5].

Rapid development of large-scale genetic variant inventories has brought attention to the identification of the genes or loci controlling phenotypic traits [6]. This triggered extensive studies on genome-wide analysis which is expected to ultimately improve our understanding in the role of unique genetic signatures for adapting environmental conditions. Recently, several genome analyses were performed to study the genetic backgrounds as well as the diversity in multiple breeds of African cattle [7–11]. For instance, a genome-wide SNP analysis for the small East African Zebu revealed the candidate loci to improve sustainable livestock productivity in the tropics [11]. Discovery of such regions in the genome enables us to detect distinct genetic variants that are related to phenotypic traits of a certain breed and facilitate functional annotation of the genome.

African trypanosomiasis is a matter of great concern that can lead to serious economic losses and health crisis in Africa. Trypanosomes are infectious agents that are transmitted by tsetse fly. It can cause lethal diseases in mammals including human and livestock. In particular, *T. congolense*, *T. vivax* and *T. brucei* groups are the main African pathogenic trypanosomes for cattle [12]. Most cattle including non-African and some African breeds (Boran, Kenana and Ogaden) are highly susceptible to trypanosome infection. Several studies have demonstrated that each breed of cattle showed an innately different degree of tolerance to trypanosomiasis when exposed to natural infection by wild-type tsetse flies from the field [13, 14]. To be specific, N’Dama breeds are naturally less susceptible to trypanosomiasis than other cattle, and hence they can survive better and maintain high productivity in trypanosomiasis-endemic areas [13, 15]. Moreover, trypanotolerant breeds including N’Dama are also less susceptible to other critical infectious diseases such as helminthiasis [13], ticks and tick-borne-diseases [3], and streptothricosis [16] in Africa. Hence, a recent study looked into trypanotolerance, one of the interesting physiological traits of indigenous African cattle. Bayesian-based method was applied to the genome data of African cattle to detect the genetic divergence that may be associated with trypanosomiasis [7]. Moreover, a systematic approach using an experimental cross between N’Dama and Boran revealed several QTLs and candidate genes controlling tolerance to trypanosomiasis in cattle [17–20].

Many studies on the tolerance to cattle trypanosomiasis-susceptibility have mainly focused on comparing N’Dama and Boran breeds. However, not many studies have carried out comparative research between N’Dama and other trypano-susceptible breeds. Herein, we concentrate on the analysis of the genetic variations between N’Dama and Ogaden cattle in order to discover N’Dama-specific genetic signatures. Ogaden cattle are one of the representative breeds that play a role as a valuable economic resource including the production of beef and dairy products, but they are known to be susceptible to trypanosomiasis [2].

In this study, a comparative genome-wide analysis of diverse cattle breeds was carried out to identify genetic distinctiveness of N’Dama breed. We investigated the genome of five indigenous African breeds and four commercial breeds using the combined methods based on information-theoretic and statistical approaches. This study identified new genetic patterns from cattle genome, and also detected selective pressures which cause an increase in genetic differentiation among populations. The proposed approaches on the analysis of the selected SNPs confirmed the differences of genomic patterns between N’Dama and other cattle breeds. Moreover, the identified associations between genes with N’Dama-specific genetic variations are related to the regulation of ossification, neurological system, and immune system development which might be involved in the evolution of N’Dama-specific phenotypes including the tolerance to African trypanosomiasis. This study reveals insights into detecting the breed-specific genetic signatures from the genome.

## Results

**Fig. 1:**
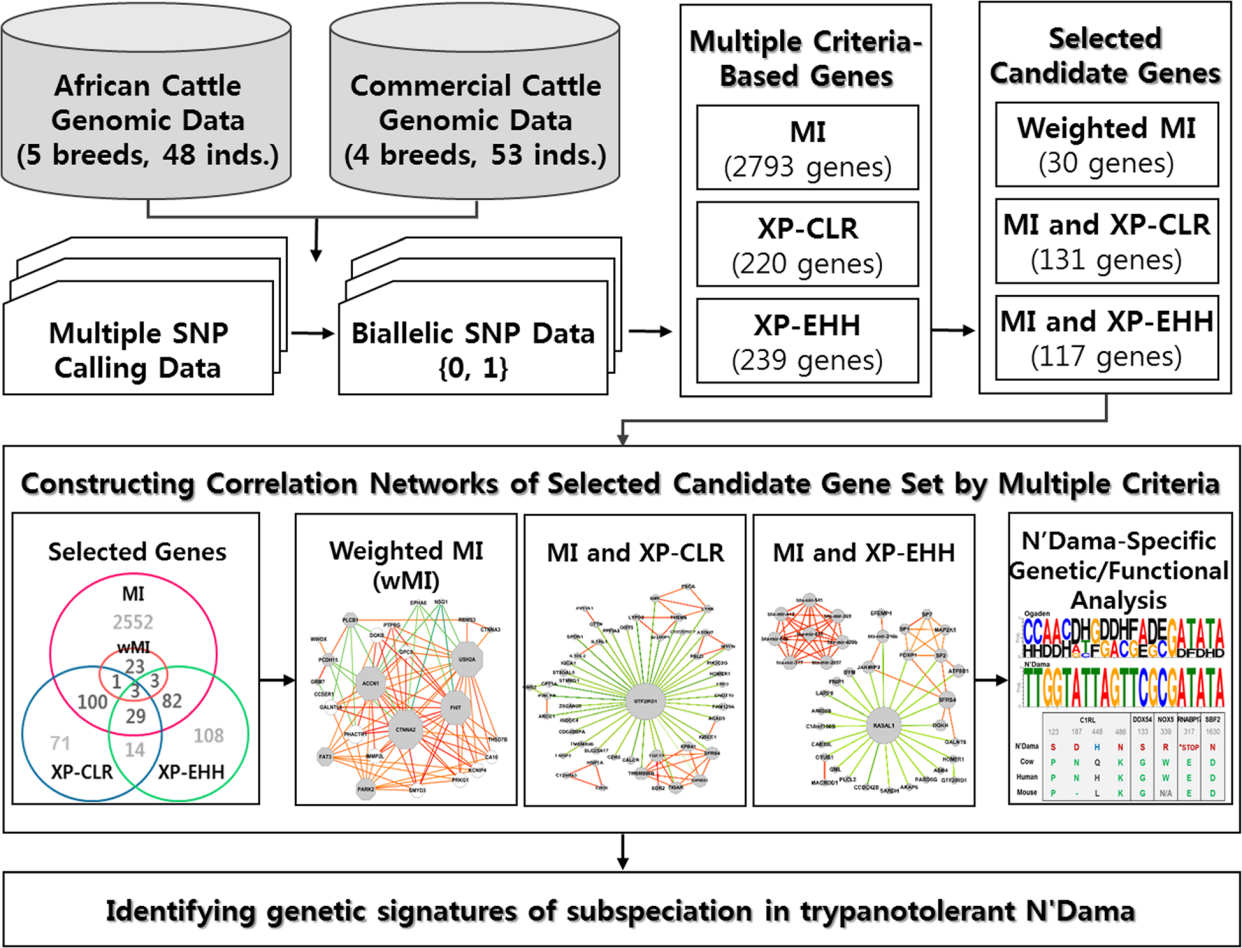
Schematic overview of systemic analysis on cattle genome for identifying genetic signatures of subspeciation in trypanotolerant N’Dama

We performed a comparative genome-wide analysis of diverse cattle breeds to discover genetic signatures of N’Dama cattle using the combined methods based on information-theoretic and statistical approaches (Fig. 1).

### Summary of sequencing, assembly and SNP detection

6.5 billion reads or ~644 Gbp of sequences with ~11 X genome coverage in total were generated from individual genomes of five indigenous African cattle (Ankole, Boran, Kenana, N’Dama and Ogaden) and four commercial cattle breeds (Angus, Hanwoo, Holstein and Jersey). The reads were aligned to the reference genome sequence UMD 3.1 with an average alignment rate of 98.84% that covered 98.56% of the reference genome (Additional file 1: Table S1). A total of ~37 million SNPs were obtained after filtering the potential PCR duplicates and correcting misalignments (Additional file 1: Table S2). Moreover, we observed 94.92% overall genotype concordance between the BovineSNP50 Genotyping BeadChip and the re-sequencing results across the samples. It helps to offer confidence on the accuracy of SNP calling (Additional file 1: Table S3).

### Identification of discriminative SNPs based on mutual information

The candidate SNPs to distinguish N’Dama and other cattle breeds were extracted using an information-theoretic method, mutual information (MI) which estimates the association strength between the SNP positions and breeds. Thus, our analysis was designed to detect the discriminative SNPs with a high dependence between the haplotypes of two adjacent loci and breeds. Approximately 2.6 hundred thousand SNPs were identified by averaging the results between N’Dama and other five breeds along with 2,793 common genes (Additional file 1: Figure S1 and S2). The extracted SNPs showed high MI values (the maximum value = 0.691) and significant *p*-values (2.13e-6). To overcome any bias caused by the small sample size, a lower *p*-value threshold was selected for estimating statistical significance (*p*-values less than 1.0e-3) compared to those in other studies [21]. Overall, these results showed that the haplotype patterns in N’Dama were clearly different from those in other cattle. Moreover, the regions containing the extracted SNPs can serve as a potential marker to distinguish N’Dama breeds.

### Difference in distribution of the SNPs identified by MI among Boran, Ogaden and N’Dama breeds

**Fig. 2:**
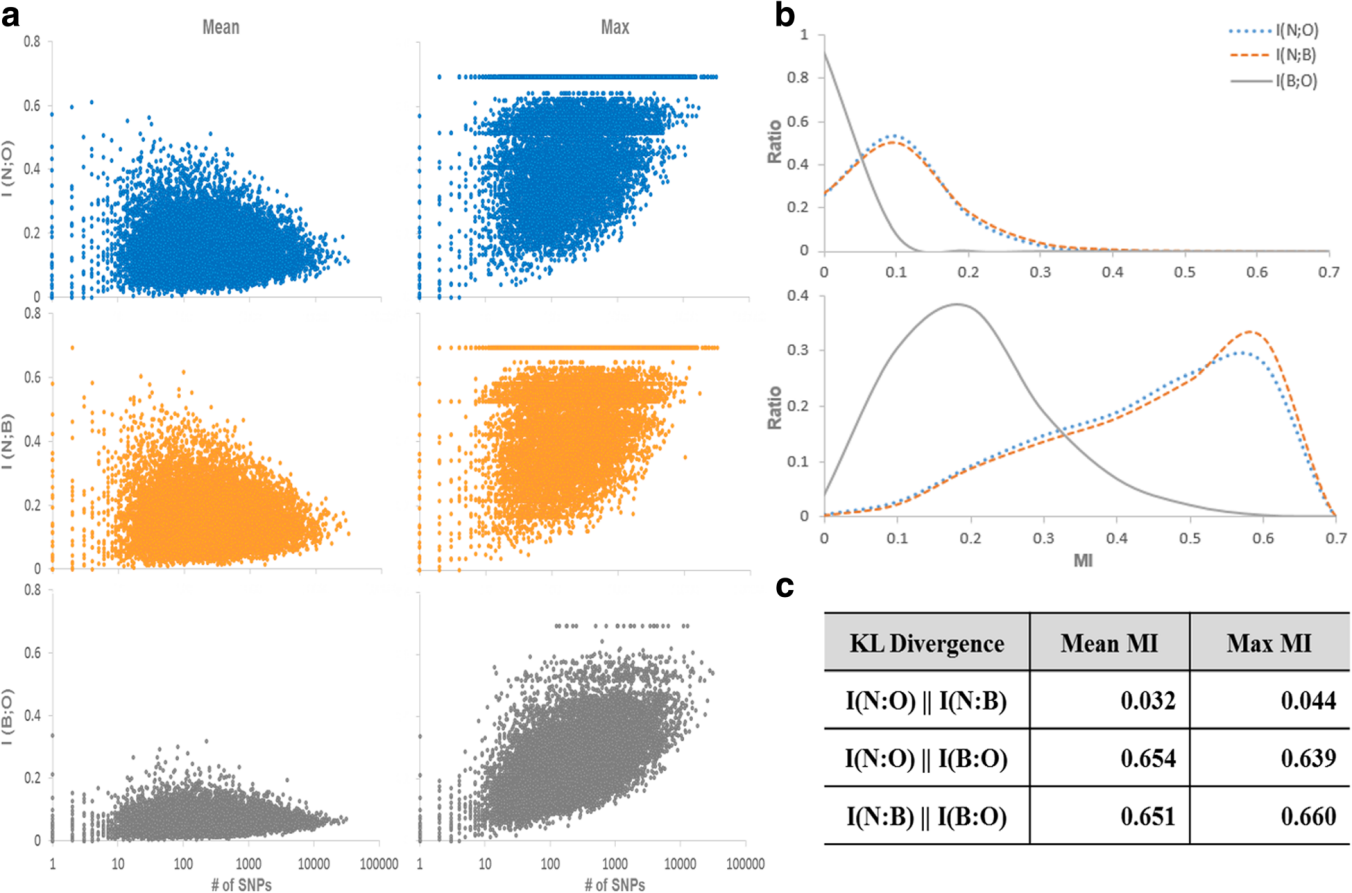
The difference in the distribution of mutual information (MI) of SNP-annotating genes between the breed pairs including Boran, N’Dama and Ogaden breed. **a** Distribution of mean and maximum values of MIs between three breed pairs on each gene is presented. All the SNPs are annotated by 16669 genes. X-axis denotes the number of SNPs annotated by a gene, and MI score is shown in y axis. Mean MI is calculated by averaging MI scores of all the SNPs annotated by a gene. Max MI is the maximum value among MI scores of all the SNPs annotated by a genes. I(N;B), I(N;O) and I(B;O) indicate MI between N’Dama and Boran, N’Dama and Ogaden, and Boran and Ogaden breed. **b** The distributions of MI ratios between Boran, N’Dama and Ogaden breed pairs. Top and bottom graphs are the ratio distribution of the mean and the max MI ratio distributions between three breed pairs, respectively. **c** The Difference in distributions between Boran, N’Dama and Ogaden breed pairs is calculated by KL divergence

The paired datasets of the three different cattle breeds including Boran, Ogaden, and N’Dama were generated as N’Dama-Boran, N’Dama-Ogaden, and Boran-Ogaden in order to identify the difference in the distribution of the identified SNPs. We computed MI values between each SNP position variable and the breed variable from the paired datasets. The total 37,363,436 SNP positions were annotated with 16,699 genes for analysing the difference of the MI distributions between N’Dama and other breeds. For the analysis, (i) the maximum, (ii) the mean, and (iii) the sum of MI values of all the SNPs in a gene were calculated in addition to (iv) the number of SNPs counted for each gene. Figure 2a shows the distributions of the mean and the maximum values of the MI of SNPs in each gene for all three pair datasets, I(N;B), I(N;O), and I(B;O). Also shown in Fig. 2a, I(B;O) values were lower compared to those of I(N;B) and I(N;O). This signifies that N’Dama breed had the SNP patterns which are distinguishable from Boran and Ogaden breeds. Such differences were likely to be associated with the unique property of N’Dama breed such as African trypanosomiasis tolerance. The differences of N’Dama from other two breeds were also clearly shown in Fig. 2b which compares the distributions of ratios for the MI values of I(N;B), I(N;O), and I(B;O). While the distributions of I(N;B) and I(N;O) were similar, those of I(B;O) clearly showed a different pattern. Considering the differential distribution of SNPs which led to the larger MI values, we suggest that N’Dama has distinctive SNP patterns which may be related to their breed-specific traits including trypanotolerance. Finally, Fig. 2c presents the Kullback-Liebler (KL)-divergence values of the MI distribution between the paired datasets of three breeds. KL-divergence is a widely used non-symmetric measure of the difference between two distributions. Larger values of KL-divergence mean larger differences between two distributions. Thus, this result also indicated that N’Dama is different from Boran and Ogaden breeds with respect to the SNP patterns which may influence N’Dama-specific traits.

### Detection of genetic signatures in N’Dama

**Fig. 3:**
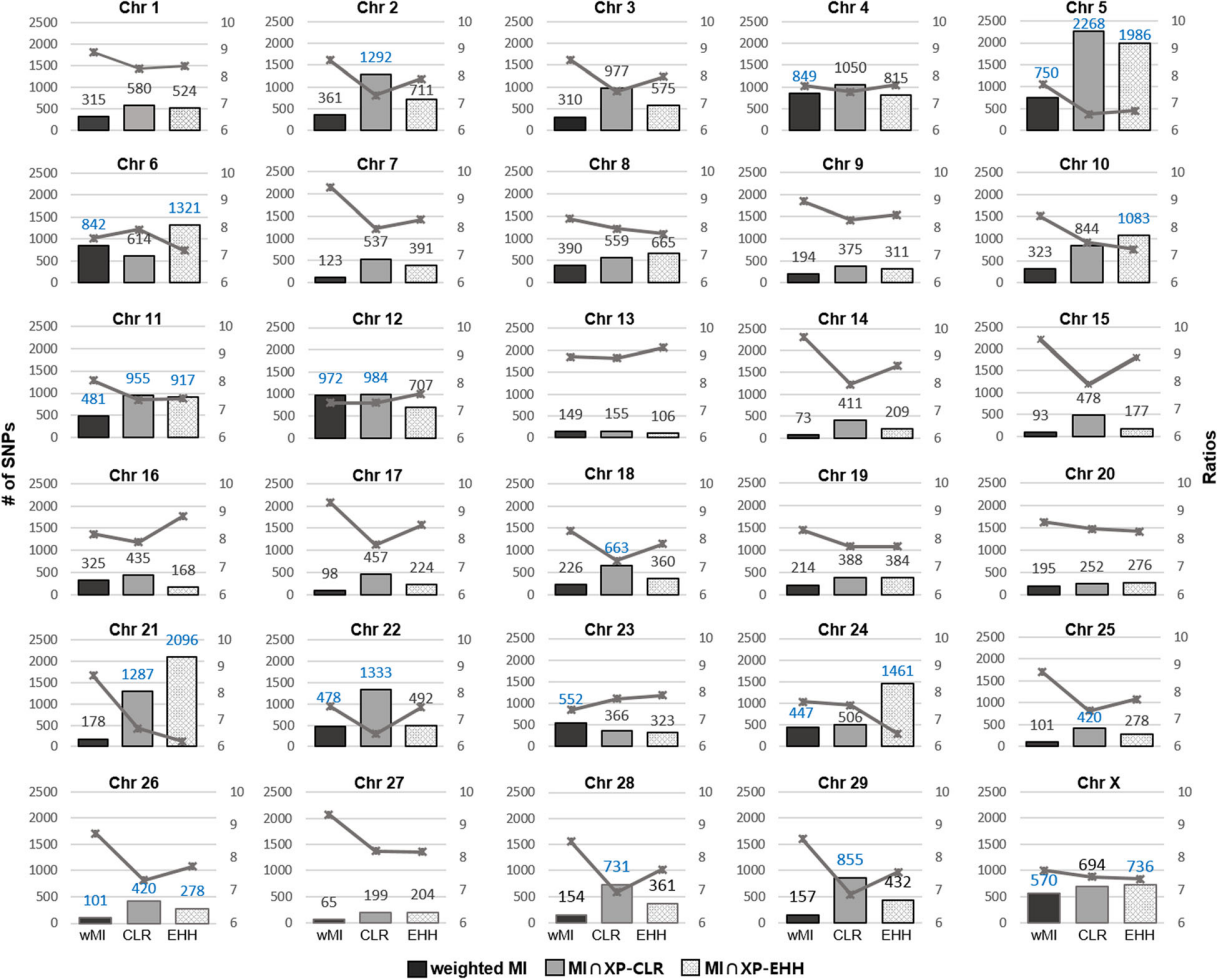
Distribution of the numbers and the log ratios of SNPs distinguishing between N’Dama and Ogaden in each chromosome. *Black*, *grey* and patterned light grey bars indicate the numbers of SNPs identified by weighted MI, the intersection of MI and XP-CLR, and MI and XP-EHH with a significant *p*-value (1.0e-2). Blue values denote the enriched chromosomes with a *p*-value less than 1.0e-2 in Fisher’s exact test. Line graphs are the ratios of the identified SNPs to total SNPs for each chromosome. The ratios are in negative log scale, thus a lower value indicates a high proportion of the SNPs distinguishing between N’Dama and Ogaden breeds. For all graphs, left y-axis represents the number of SNPs and right y-axis indicates the ratio value

We performed the analysis with the weighted mutual information (wMI) in order to scan the genome for breed-specific SNPs. For a given gene, wMI is defined as the summation of two factors: the normalized number of SNPs assigned to the gene and the mean MI value of SNPs of the gene. The proposed wMI is considered as the degree of the genetic variation in the gene and as the discriminative information between the breeds. Figure 3 shows the distribution of the significant SNPs identified by wMI across all 30 chromosomes as well as the intersection of MI and XP-CLR, and MI and XP-EHH on each chromosome, and the degree of enrichment in each chromosome with Fisher’s exact test. Fisher’s exact test was performed with a 2×2 contingency table, composed of two factors: whether the SNP is included in a specific chromosome, and whether the SNP is identified by each measure. We also presented the distribution of the genes including significant SNPs identified by the same three measures for each chromosome (Additional file 1: Figure S3). Although SNPs were found in all chromosomes, the number of the SNPs were not even across the chromosomes. Especially, when the intersection of MI and XP-CLR, and MI and XP-EHH were applied, relatively large number of SNPs were detected in chromosome 5. These distributions of the SNPs on each chromosome provided the information on genomic locations that are likely to have received selection pressure and possess the ability to distinguish N’Dama and Ogaden breeds.

### N’Dama-specific SNPs identified by wMI

Thirty genes containing the distinctive SNPs between N’Dama and Ogaden were identified by wMI analysis (Additional file 1: Table S4). We constructed correlation networks with the identified genes. The networks were generated based on the correlation coefficients of the gene variation degrees which are obtained by calculating the variations of SNPs annotated by each gene. The SNP variation is the difference between alleles of the same SNP position for cattle samples. It indicates the degree of homozygosity or heterozygosity of SNPs which is defined as the ratio of homozygous or heterozygous alleles for all samples of a breed. For instance, when the allele pair of SNP_1 of most samples of breed_1 is “AA”, the homozygosity of SNP_1 for breed_1 is large. The heterozygosity of SNP_1 for breed_2 is high when SNP_1 allele pair of most breed_2 samples is “AT”.

**Fig. 4:**
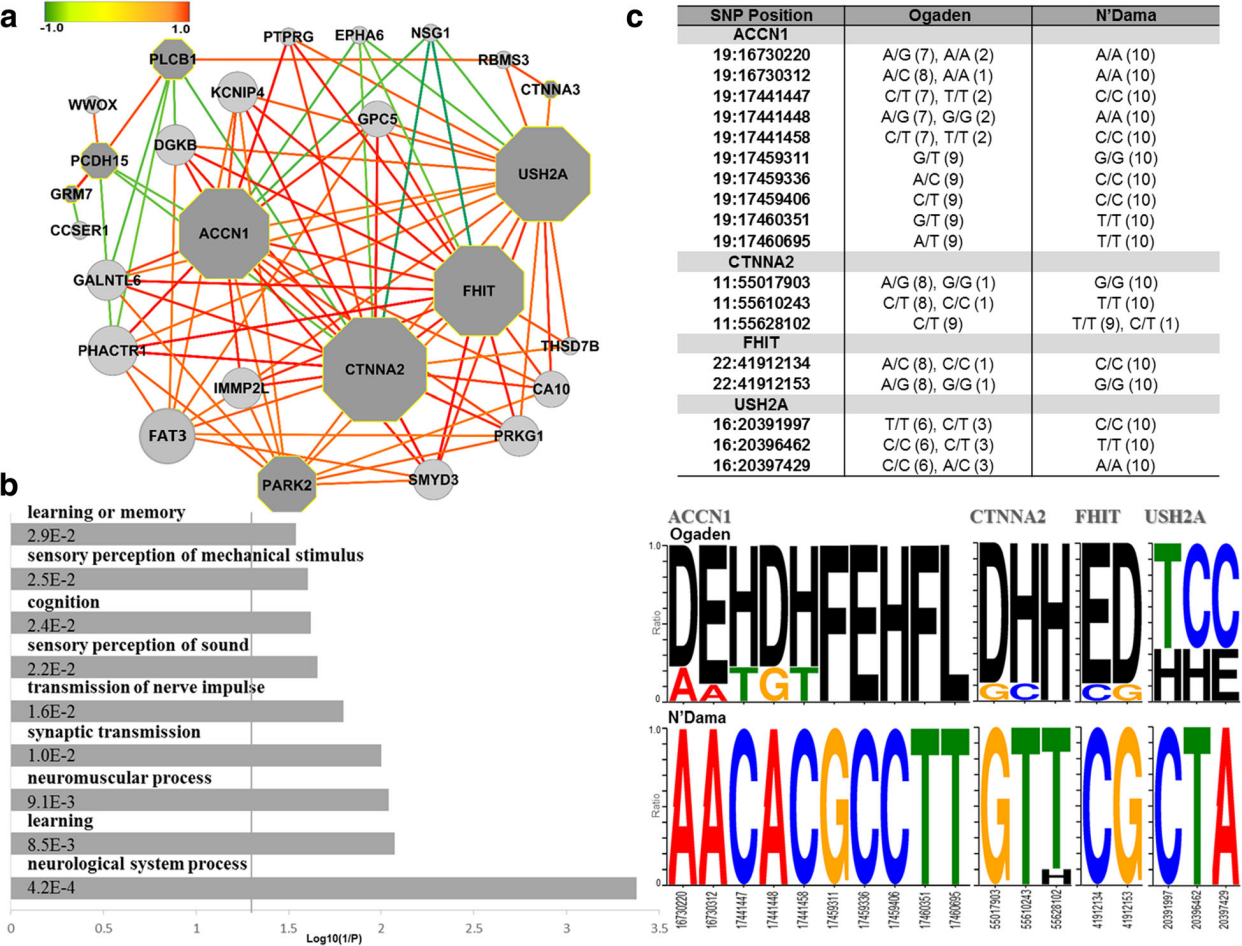
Genes identified based on the wMI for N’Dama breed. **a** The correlation network of 30 genes selected using the wMI of SNP types between N’Dama and Ogaden breeds. Circles denote genes including SNPs identified by the wMI and dark grey octagons represent the genes annotated in the result of GO analysis. Dark *red* edges indicate strong positive correlations and dark *green* edges show strong negative correlations (gene pairs with a correlation coefficient value larger than 0.45 or smaller than -0.25 are connected). **b** GO analysis on the extracted genes by adjusting thresholds from the constructed correlation network. A vertical line indicates an FDR-adjusted *p*-value (0.05). **c** The genotype profiles of the identified genes including ACCN1, CTNNA2, FHIT, and USH2A are shown. The SNP positions of each gene reveal clearly different patterns between N’Dama and Ogaden breeds. Each logo indicates A (A/A), T (T/T), G (G/G), C (C/C), L (A/T), D (A/G), E (A/C), F (G/T), H (C/T), and I (C/G). Upper table shows the types of SNP alleles for each breed. Values in the parentheses represent the numbers of samples with each allele for four genes

The constructed network showed that ACCN1, CTNNA2, FHIT and USH2A function as main hubs of the network (Fig. 4a). The heterozygosity or homozygosity of SNPs in many genes of the network was strongly associated with that in these four genes. ACCN1 encodes a sodium channel protein which is expressed in both the central and peripheral nervous system. It regulates neuronal activity in a pH-dependent manner. The diverse physiological roles of ACCN1 in neuronal systems include synaptic plasticity, learning, fear, pain sensation, mechanosensation, and neurodegenerative diseases [22]. CTNNA2 is known as a linker between cadherin receptors and the cytoskeleton to regulate cell-cell adhesion and differentiation in the nervous system, and is implicated in several neurological functions including the control of startle modulation [23]. FHIT protein is a member of the histidine triad gene family of nucleotide hydrolases involved in purine metabolism. This gene contributes to the regulation of gene expression essential for cell proliferation and survival and tumor suppressor [24]. USH2A is found in the basement membrane of the cochlea and the retina, and is believed to take part in adhesion of pre- and post-synaptic membranes and in nerve fiber guidance. Mutations in the USH2A gene are also responsible for a subtype of Usher syndrome which is the most frequent cause of combined deaf-blindness in man [25].

In addition, we performed GO analysis with the genes extracted by the threshold of correlation coefficient (larger than 0.8 or smaller than -0.3) in the constructed network. Enriched terms were related to cognitive functions (‘learning’, ‘learning or memory’ and ‘cognition’), perceptual systems (‘sensory perception of sound’ and ‘sensory perception of mechanical stimulus’) and neurological systems (‘neurological system process’, ‘neuromuscular process’, ‘synaptic transmission’, ‘sensory perception of mechanical stimulus’, and ‘transmission of nerve impulse’) (FDR adjusted *p*-value < 0.05) (Fig. 4b; Additional file 1: Table S5). This result strongly indicated that N’Dama may be distinguished from the other breeds of African cattle by a neurological system related to startling response which requires sensory perception, learning or memory as well as neuromuscular system. Furthermore, Fig. 4c displayed genotype profiles for each SNP position on the above-mentioned four genes. Interestingly, the genotypes of the identified SNPs revealed different patterns between N’Dama and Ogaden breeds. Genotypes of N’Dama were biased for homozygosity and were found to be more homogeneous within the population than those in Ogaden.

### N’Dama-specific SNPs identified by MI and XP-CLR

**Fig. 5:**
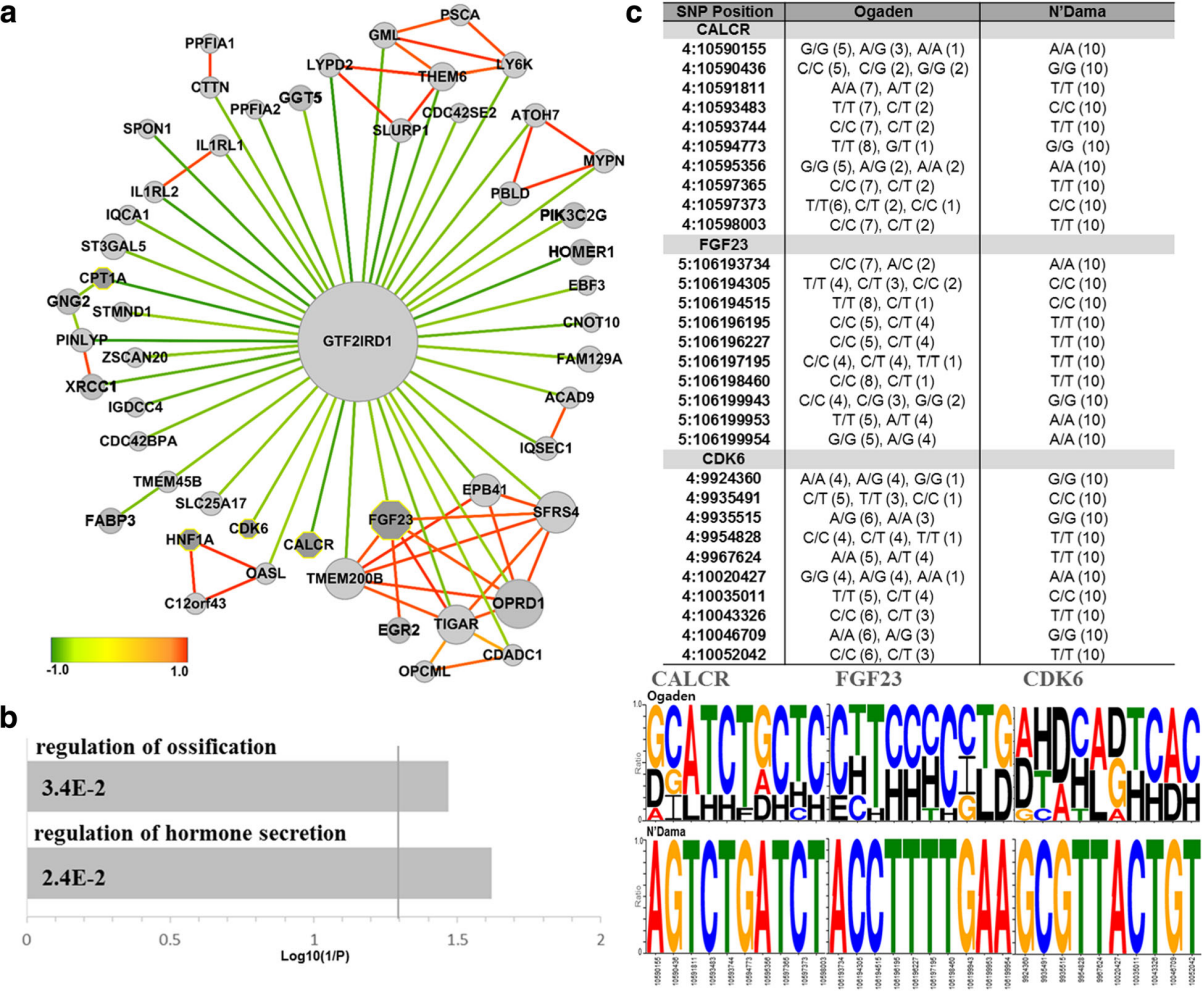
Genes identified based on the MI and XP-CLR for N’Dama breed. **a** The correlation network of 131 genes selected using the MI and XP-CLR of SNP types between N’Dama and Ogaden breeds is presented. Circles denote genes identified by the intersection of MI and XP-CLR and dark grey octagons represent the genes annotated in the result of GO analysis. Dark *red* edges indicate strong positive correlations and dark *green* edges are strong negative correlations (gene pairs with a correlation coefficient value larger than 0.9 or smaller than -0.4 are connected). **b** GO analysis on the extracted genes by adjusting thresholds from the constructed correlation network is shown in this figure. A vertical line is an FDR-adjusted *p*-value (0.05). **c** The genotype profiles of the identified genes, CALCR, FGF23 and CDK6, for 10 representative SNP positions of each gene clearly show different patterns between N’Dama and Ogaden breeds. Upper table reveals the types of SNP alleles for each breed. Values in the parentheses denote for the numbers of samples with each allele for the three genes

In the next step, we identified genes displaying genetic signatures which may have contributed to the development of N’Dama-specific phenotypes. Two gene lists were created one of which containing 2,793 genes obtained from MI analysis and the other containing 220 genes from XP-CLR. The 131 genes found in common between these two lists represented a set of functional genes that facilitated adaptation of N’Dama to the local environment (Additional file 1: Table S6). A correlation network based on the identified genes demonstrated that the genotype of SNPs in many genes were negatively associated with a single hub gene known as general transcription factor or GTF2IRD1 (Fig. 5a). GTF2RD1 has been intensely studied in brain and embryo due to its involvement in a rare neurodevelopmental disorder, Williams-Beuren syndrome [26]. Chimge *et al.* [27] observed overexpression of GTF2RD1 in mouse embryonic fibroblast cells, and reported that GTF2IRD1 regulates many genes that are involved in a variety of biological processes such as immune response, cell cycle, cell signaling and transcriptional regulation. The expression levels of ATOH7, IL1RL2, OASL and OPRD1 changed after GTF2RD1 overexpression [27]. When a SNP type is defined based on the degree of the heterozygosity or homozygosity of SNPs for all samples, the associations of SNP types between GTF2IRD1 and those mentioned target genes were also observed in our correlation network using the combined measure of the MI and the XP-CLR. These results may reflect modified biological interactions of GTF2IRD1 with target genes in N’Dama as opposed to other African cattle and commercial breeds. In addition, the genotype profiles of this gene showed differences between N’Dama and Ogaden breeds (Additional file 1: Figure S4).

We also carried out GO analysis with the genes selected by the threshold of correlation coefficient (larger than 0.97 or smaller than -0.5) in the constructed network. The significantly enriched terms included ‘regulation of hormone secretion’ and ‘regulation of ossification’ (FDR adjusted *p*-value < 0.05) (Fig. 5b; Additional file 1: Table S7). The terms that were related to appearance of the ossification enriched by genes including CALCR, FGF23, and CDK6 suggest pathways that may provide deeper insights into understanding some aspects of the N’Dama-specific features. In particular, CALCR is a high affinity receptor for the peptide hormone calcitonin. This receptor is known to be associated with maintaining calcium homeostasis enhancing calcium excretion by the kidneys and it also takes part in regulating osteoclast-mediated bone resorption [28]. FGF23 is a regulator of phosphate homeostasis and vitamin-D metabolism. This protein is reported to negatively regulate osteoblast differentiation and matrix mineralization [29]. Finally, CDK6 which is a member of a protein kinase is an important regulator of cell cycle progression. It also prevents myeloid differentiation by interfering with RUNX1, a transcription factor that regulates the differentiation of hematopoietic stem cells into mature blood cells [30]. Furthermore, we identified IL1RL1 and IL1RL2 in the constructed network in concordance with the observation that the initial response of the host immune system to trypanosomes infection contains the activation of macrophages secreting pro-inflammatory molecules such as IL-1 [31, 32]. In particular, it has been previously reported that *T. brucei* infections lead to the increase of IL-1 secretion [33]. Apart from the GO analysis, we showed that N’Dama and Ogaden possess distinct patterns of homozygosity and heterozygosity for the SNP alleles of CALCR, FGF23, and CDK6 (Fig. 5c). Taken together, these results indicated that genetic diversification has occurred between N’Dama and Ogaden, in the genes related to the regulation of ossification.

### N’Dama-specific SNPs identified by MI and XP-EHH

**Fig. 6:**
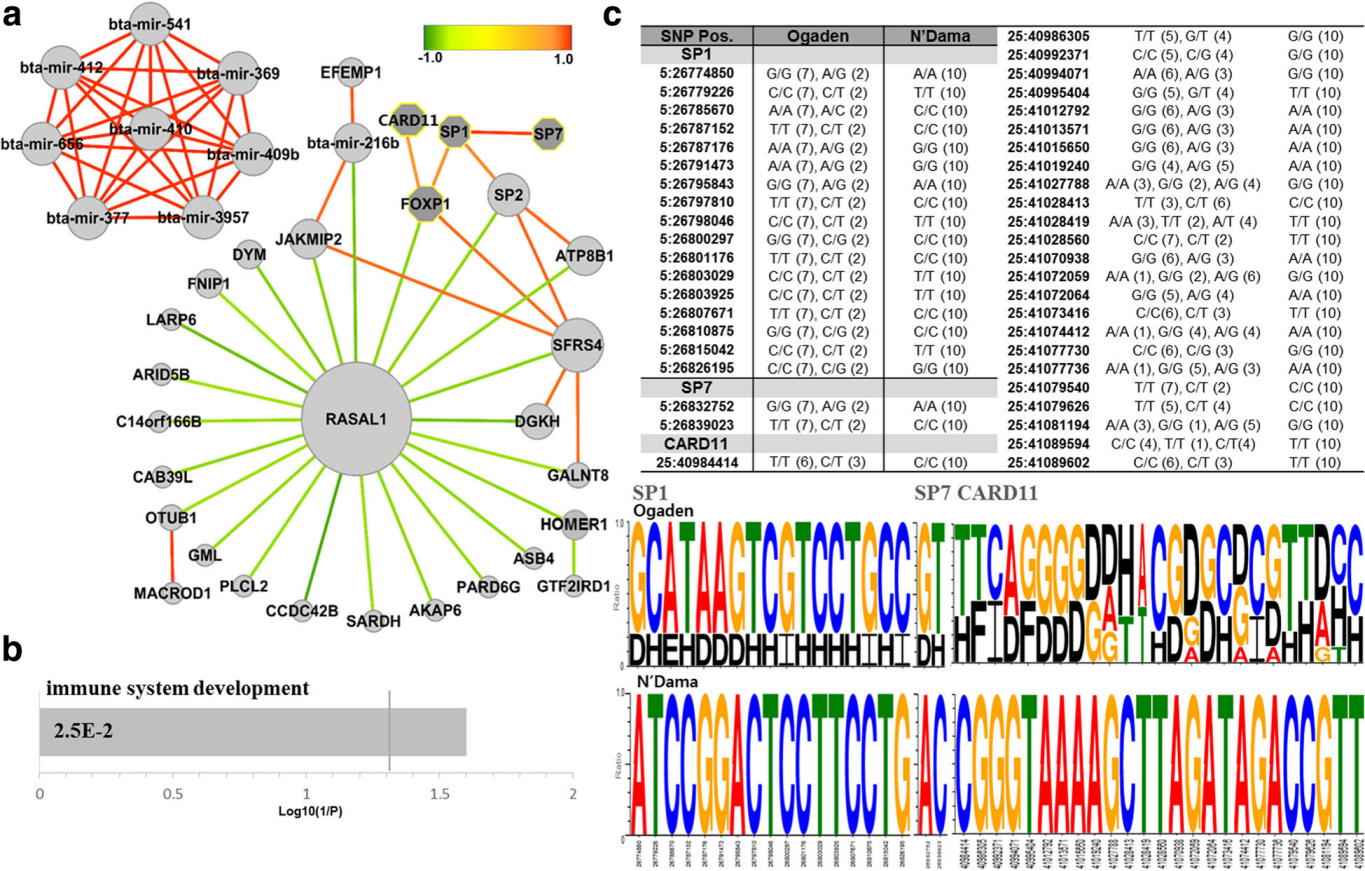
Genes identified based on the MI and XP-EHH for N’Dama breed. **a** Correlation network of 117 genes selected using the MI and XP-EHH of SNP types between N’Dama and Ogaden breeds is demonstrated. Circles denote genes identified by the intersection of MI and XP-EHH and dark grey octagons represent the genes annotated in the result of GO analysis. Dark *red* edges indicate strong positive correlations and dark *green* edges are strong negative correlations (gene pairs with a correlation coefficient value larger than 0.9 or smaller than -0.5 are connected). **b** GO analysis on the selected genes by adjusting thresholds from the constructed correlation network is presented (excluding miRNAs). A vertical line represents an FDR-adjusted *p*-value (0.05). **c** The genotype profiles of the identified genes, SP1, SP7 and CARD11, for each SNP position clearly reveal different patterns between N’Dama and Ogaden breeds. Table shows the types of SNP allele for each breed. Values in the parentheses represent the numbers of samples with each allele for three genes

The 117 common genes were identified in the lists of 2,793 genes from MI and 239 genes from XP-EHH (Additional file 1: Table S8). The correlation network analysis performed on those genes showed that the genotypes of SNPs in many of these genes were negatively related to a hub gene, RASAL1 (Fig. 6a). RASAL1 is a member of ras GTPase-activating protein families and recently reported to be a tumor suppressor gene in several types of cancer [34, 35]. The SNP alleles of RASAL1 in N’Dama also represented homozygous types unlike Ogaden breeds (Additional file 1: Figure S5).

GO analysis of the genes extracted by the threshold of correlation coefficient (larger than 0.97 or smaller than -0.5) in the constructed network showed significantly enriched terms, ‘immune system development’ (FDR adjusted *p*-value < 0.05) (Fig. 6b; Additional file 1: Table S9). CARD11, FOXP1 and SP1 were significantly over represented in ‘immune system development’. In particular, CARD11 is critical for signaling in T- and B-lymphocytes in both the innate and adaptive immune system, and it transmits signals from antigen receptors to the transcription factor NF-kB [36, 37]. FOXP1 belongs to subfamily P of the forkhead box (FOX) transcription factor family which plays important roles in the regulation of tissue- and cell type-specific gene transcription during embryo development and adulthood. More specific function of FOXP1 includes the regulation of cardiomyocyte proliferation [38], motor neuron development [39], and B-cell development [40]. In addition, similar to the result from the analysis of MI and XP-CLR, ossification-related terms were enriched with significant *p*-values (the modified Fisher exact *p*-value < 0.05) due to genes including SP1 and SP7 (Additional file 1: Table S9). SP1 is a zinc finger transcription factor involved in many cellular processes including cell differentiation, apoptosis, immune responses, and osteogenic differentiation of dental stem cells [41]. On the other hand, SP7 is a bone-specific transcription factor that is required for the activation of a range of genes during osteoblast differentiation and bone formation [42]. Also, it was reported by other studies that some of SP7-expressing osteoblast precursors travel through the cartilage template and form stromal cells in the bone marrow space in which hematopoiesis occurs [43, 44]. Fig. 6c presents the SNP profiles for SP1 and SP7 genes between N’Dama and Ogaden breeds. Two genes showed the opposite zygosity SNP pattern in N’Dama and Ogaden respectively. These results imply that SNP variants may be involved in the gene regulation between N’Dama and Ogaden breeds.

Furthermore, we also observed that majority of SNPs found in eight miRNAs (bta-miR-369, bta-miR-377, bta-miR-409b, bta-miR-410, bta-miR-412, bta-miR-541, bta-miR-656, and bta-miR-3957) showed homogeneity in SNP variation (Fig. 6a). Notably, these miRNAs are located in close proximity to one another in chromosome 21 between 67,598,000 and 67,604,800 and five of which including bta-miR-369, -377, -409b, -410 and -656 are the members of miR-154 family. Homologs of miR-154 family found in human are originally known to be overexpressed in idiopathic pulmonary fibrosis [45]. In addition, recent evidence suggests the association of the function of this miRNA family with the bone development. Li *et al*. [46] reported that expression of miR-410 and miR-154 are decreased in tension-treated adipose-derived mesenchymal stem cells (ADSCs), and that miR-154 inhibits osteogenic differentiation of ADSCs through the WNT/PCP pathway by directly regulating WNT 11. This result indicated that the SNP variants may cause differential expression of miRNA which in turn influence expression of their target genes between N’Dama and Ogaden breeds.

### Identification of N’Dama-specific missense and nonsense mutations

Finally, we looked into variations on a protein level by focusing onto non-synonymous SNPs and investigated whether such variations caused any physiological change in N’Dama cattle. N’Dama-specific missense or nonsense variants with their annotated genomic locations and coding effects for the identified genes were observed after performing the three measures (Additional file 1: Table S10). All missense or nonsense mutations observed were summarized in Table 1: 20 missense mutations in 15 protein coding genes, and a nonsense mutation with one variants in RANBP17. Many of the annotated genes are associated with immune (C1RL, EOMES and TPST1), nervous (AMZ1, DDX54, EML1, OPCML, SBF2, SLIT3 and USH2A) and cellular metabolic (ACAD9, CDADC1, NOX5 and TIGAR) systems. The gene description and the related function for each gene are shown in Additional file 1: Table S11.

**Table 1:** List of the identified genes including N’Dama-specific missense and nonsense mutations. AA, amino acid

Proposed model	Gene	Transcript ID	Location	Mutation DNA	Mutation AA / Total length	Property change of AA	Domain (Interpro)	*P*-value
wMI	USH2A	ENSBTAT00000061112	16:19646967	CTG/GTG	L4574V / 5204	Non polar aliphatic	Fibronectin type III	4.3E-11
MI ∩ XP-CLR	ACAD9	ENSBTAT00000050396	22:59610742	TGG/CGG	W520R / 565	Hydrophobic aromatic → Basic	-	1.0E-11
	AMZ1	ENSBTAT00000037103	25:41186216	GTC/ATC	V204I / 499	Non polar aliphatic	-	1.7E-15
			25:41189837	GAC/AAC	D114N / 499	Acidic → Polar uncharged	-	1.4E-11
	CDADC1	ENSBTAT00000002932	12:18986879	ACT/CCT	T480P / 516	Polar uncharged → Hydrophobic aromatic	Deoxycytidylate deaminase-related	1.3E-11
	EML1	ENSBTAT00000017944	21:66542615	GTC/ATC	V589I / 823	Nonpolar aliphatic	-	3.1E-11
	EOMES	ENSBTAT00000061448	22:2116678	GGG/AGG	G255R / 682	Nonpolar aliphatic → Basic	-	7.7E-12
	OPCML	ENSBTAT00000030247	29:35039075	GAA/AAA	E53K / 103	Acidic → Basic	H4	7.3E-12
	PIK3C2G	ENSBTAT00000043102	5:92276870	TTC/GTC	F24V / 1487	Hydrophobic aromatic → Nonpolar aliphatic	-	5.2E-13
			5:92276593	CCA/CAA	P116Q / 1487	Hydrophobic aromatic → Polar uncharged	-	2.2E-10
	SLIT3	ENSBTAT00000049620	20:397494	ACC/AGC	T605S / 1339	Polar uncharged	Leucine-rich repeats	1.3E-10
	TIGAR	ENSBTAT00000022146	5:106225048	AAA/AGA	K91R / 270	Basic	Histidine phosphatase superfamily	2.0E-11
	TPST1	ENSBTAT00000000502	25:28371859	ATG/ACG	M235T / 370	Hydrophobic Aliphatic → Polar uncharged	P-loop containing nucleoside triphosphate hydrolase	1.7E-12
MI ∩ XP-EHH	C1RL	ENSBTAT00000021566	5:103639896	CCC/TCC	P123S / 487	Hydrophobic aromatic → Polar uncharged	CUB domain	4.3E-11
			5:103641644	AAC/GAC	N187D / 487	Polar uncharged → Acidic	-	4.3E-11
			5:103644203	CAG/CAC	Q448H / 487	Polar uncharged → Basic	Serine proteases, trypsin domain	5.2E-13
			5:103644317	AAG/AAC	K486N / 487	Basic → Polar uncharged	-	6.4E-13
	DDX54	ENSBTAT00000029525	17:63462022	GGC/AGC	G133S / 877	Nonpolar Aliphatic → Polar uncharged	DEAD/DEAH box helicase domain	3.1E-12
	NOX5	ENSBTAT00000011888	10:15844849	TGG/GGG	W339G / 755	Hydrophobic aromatic → Nonpolar aliphatic	Ferric reductase transmembrane component-like domain	3.3E-16
	RANBP17	ENSBTAT00000030757	20:3004702	GAG/TAG	E54* / 317	*STOP	Transposase, type 1	7.8E-11
	SBF2	ENSBTAT00000061615	15:43473534	GAT/AAT	D1630N / 1848	Acidic → Polar uncharged	Myotubularin family	1.1E-14

Also, the 15 mutations out of 20 missense mutations resulted in alteration of chemical properties. Eleven mutations were located in functional domains, while the rest nine were in inter-domain region (Table 1). AMZ1, C1RL and PIK3C2G exhibited multiple protein mutations. Even though these mutations were not found within the functional domains, amino acid properties were changed. Notably, C1RL displayed four mutations, all of which resulted in altered properties of amino acids. Two mutations including CUB and trypsin-like serine protease domain were located in the functional domains. Several proteins containing CUB and trypsin-like serine protease domains are associated with complement activation, tissue remodeling and cellular migration. It has been suggested that C1RL is involved in complement pathways during inflammation although its physiological role is not well-understood [47]. We also found one nonsense variant (rs385712825) with a significant *p*-value (7.82e-11). This SNP was located in RANBP17 which is a member of the importin-*β* super family of nuclear transport receptors. In human, RANBP17 is the loci of recurrent chromosomal 5 breakpoints detected in T-cell acute lymphoblastic leukemia, and the transcriptional activation of this gene occurs during hematopoietic process with enhancer elements of the TCR delta gene [48].

**Fig. 7:**
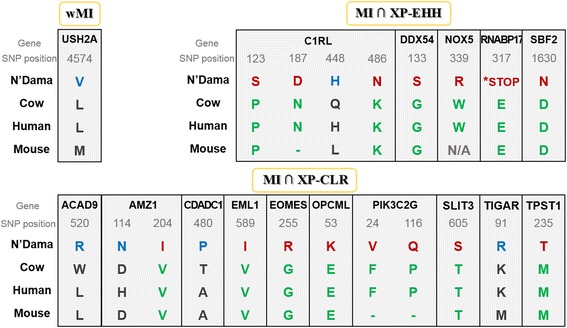
Amino acid substitution resulted from the missense and nonsense mutations of the genes identified by wMI, the intersection of MI/XP-CLR and MI/XP-EHH. The 20 missense and one nonsense variants of the identified genes show distinguishing amino acid substitution in N’Dama compared to that of reference cow (UMD 3.1), human and mouse

Furthermore, we compared the amino acids encoded by the 20 missense and one nonsense mutations in N’Dama with the corresponding amino acids in reference cow (UMD 3.1), human and mouse (Fig. 7). Interestingly, the amino acids substitution in the variant positions were detected only in N’Dama which clearly distinguished N’Dama from other cattle breeds and species. It implies that the mutated alleles affected coding changes leading to alterations in the function of the identified genes.

## Discussion

The development of large-scale genetic variant inventories has triggered a number of studies on the identification of distinct genome patterns which give rise to breed-specific traits. For instance, several researches attempted to detect genetic divergences that are associated with trypanosomiasis in African cattle from genome data [7, 10]. In this study, a genome-wide comparative analysis was performed with SNP data from various cattle breeds, including African indigenous cattle and commercial breeds, in order to identify the genetic signatures of N’Dama.

Comparison of N’Dama genome with other indigenous African cattle and commercial breeds resulted in the identification of N’Dama specific SNPs. MI analysis for the detection of breed-specific SNPs successfully distinguished genotypic profiles among Boran, N’Dama and Ogaden. In addition, the combination of either MI and XP-CLR or MI and XP-EHH allowed us to screen positively selected SNPs in N’Dama genome that are presumed to have occurred during natural and artificial selection. Genetic regions uncovered by XP-EHH and XP-CLR often represent biologically meaningful variations that may explain adaptive traits. Moreover, it is possible to produce larger lists of likely selective sweeps, and as a result, this may allow us to better understand how selection has affected the variation of a specific-breed [49]. Some of the positively selected SNPs located in genic region were unique in N’Dama when compared to commercial breed and other mammals. Furthermore, some variants in N’Dama were homogeneous, and these N’Dama-specific variants were also detected in the pool of Ogaden genotypes. Ogaden possessed not only more heterogeneous but also bigger genetic pools than N’Dama. Numbers of detected SNPs were significantly high in some of the chromosomes (*p*-values less than 1.0e-2), indicating greater selection pressures to these chromosomes during the evolutionary history of N’Dama.

The correlation network is constructed based on the similarity of genotype between genes. If a SNP variation value at gene level is close to 0, this means that the gene possesses similar genotypes to the reference. On the other hand, if the SNP variation at gene level is higher, the gene is likely to possess relatively more heterozygous or alternative homozygous genotypes. The interaction between genes in the correlation networks shows similarity in their genotypes. If calculated trends of genotypes for two genes are homo-homo or hetero-hetero, their correlation will be high (close to 1) and the edge will be red as shown in the Fig. 4a, 5a and 6a. Whereas, if the collective genotype is homo for one gene and hetero for the other gene, then the correlation will be low (close to -1) and the edge will be in green. In the correlation network, hub genes connected by negative correlation edges can be differently interpreted from hub genes with positive edges. Since a hub node is usually important in many networks, highly connected hub genes are expected to play a significant role in biological networks [50]. Thus, the hub genes we found are expected to have potentials for distinguishing between N’Dama and Ogaden. In particular, two negative hub genes including GTF2IRD1 and RASAL1 can be considered as genes with the opposite zygosity against most of the node genes. We speculate that the homo- or hetero- zygosity of two genes is likely to play a distinct role from other genes. It can be expected to provide an opportunity to formulate potential hypotheses for investigating biological processes.

Comparison of genomes among different cattle breeds using wMI identified statistically significant SNPs and the genes where these SNPs are located. From the analysis based on wMI approach, many genes of the constructed network and the majority of enriched GO terms indicated that N’Dama may have a distinguished sensory and neurological system related to startle response (Fig. 4b; Additional file 1: Table S5). Notably, the investigation of the acoustic startle response in terms of brain and genetic mechanisms revealed the involvement of genetic factor [51, 52]. For example, there are a wide range of responses across the inbred strains in rodents [53–55]. It is plausible that indigenous African cattle may possess various levels of startling and fear responses. The mammalian startle response is related to defence system and plays a critical role in survival of the species throughout evolution [56]. In addition, fear has greatly affected the process of animal domestication, especially when animals become frightened of the people who handle them [57]. This imply that unique neuronal circuitries of startle response and cognition might play a critical role in specification, adaptation, and domestication of N’Dama cattle. Unfortunately, not many studies on neurology of normal or trypanosomiasis infected N’Dama cattle exist. Hence, the functional consequences and pathogenic relevance of the neurological features regarding trypanotolerance remain to be elucidated. Although we could not directly associate N’Dama-specific neurological features with trypanotolerance, these results may be considered as genetic signatures distinguishing N’Dama from the other cattle breeds due to their statistical significance.

While wMI methods extracted statistically significant SNPs by comparing genomes of different breeds, XP-EHH and XP-CLR detected SNPs that were influenced by positive selection. Both the combined MI and XP-CLR, and MI and XP-EHH analysis identified genes involved in ossification. This may reflect the differences in feed efficiency and growth traits between N’Dama and Ogaden which may result in smaller skeletal size of N’Dama. Additionally, N’Dama has developed superior ability to survive under unfavourable environment while Ogaden has been selected for better dairy and beef production. In many genome-wide association study (GWAS) and genomic predictions for feed efficiency and growth traits in commercial beef and dairy cattle, the positive or negative regulation of ossification and bone mineralization is commonly observed in relation to traits like average daily gain, or mid-test metabolic weight [58, 59].

Enrichment in the term “ossification” may also indicate physiological difference between N’Dama and Ogaden. Ossification has several functions: for instance, skeletal growth, mineral storage, blood cell production, and energy storage. The genes associated with ossification were implicated in biological process such as calcium homeostasis (CALCR), phosphate homeostasis and vitamin-D metabolism (FGF23), cell cycle progression (CDK6), and the regulation of transcription (SP1 and SP7) involved in multiple functions (osteogenic formation, bone formation, differentiation, apoptosis, and immune response).

According to previous studies on trypanosomiasis, responses to trypanosoma infection in cattle include immunosuppression, inflammatory response and anaemia [17, 19, 60]. CARD11 over-represented in ‘immune system development’ plays important roles in innate and adaptive immune system, and contributes to NF-kB activation in various signalling cascades [36, 37]. The activation of NF-kB is known as a determinant of the intracellular survival and tissue tropism of *T. cruzi* that causes human sleeping sickness [61]. This may suggest that CARD11 affecting NF-kB activation is possible to change in functions to effectively control the infection of *T. brucei*. In addition, haematopoietic stem cells (HSCs) in bone marrow give rise to the different types of mature blood cells and immune cells. Our results imply that N’Dama may possess specific genetic factors that confer immunity to supress activities of trypanosomes more effectively. A previous genome-wide study performed with West African cattle revealed that genes involved in immune response were under strong balancing selection in trypanotolerant N’Dama breeds [7] which also supports the implication suggested by our result. Furthermore, bone marrow function and blood cells have been suggested to take parts in the development of trypanosomiasis [62–64].

Through examining exonic SNPs that results in missense or nonsense mutation (Table 1 and Additional file 1: Table S10), we identified three main biological processes associated with the immune system (Additional file 1: Table S11). All of the mutations were specific in N’Dama cattle compared to other cattle, mouse and human (Fig. 7). Although these mutations are required to be validated for functional and physiological consequences in the future studies, we suggest that the biological processes related to immunity may be a part of the strong candidate systems that give rise to trypanotolerance.

## Conclusions

In conclusion, our results illustrate that trypanotolerant N’Dama displays clear genetic differences compared to other African cattle and commercial breeds. The adaptation of N’Dama to the environment may implicate unique bone formation related to growth traits, immuno-genetic mechanisms that allow them to tolerate regional diseases including trypanosomiasis, and neurological processes which involved in the development of favorable behaviors for survival. Our analysis provides advanced knowledge in genetic selection of N’Dama and its adaptation to the local environment.

## Methods

### Samples, DNA resequencing and SNP detection

Whole-blood samples (10 ml) were collected from indigenous African cattle (10 Ankole, 10 Boran, 9 Kenana, 10 N’Dama, 9 Ogaden breeds) and commercial cattle (10 Angus, 10 Jersey, 10 Holstein and 23 Hanwoo breeds). The DNA was isolated from the whole blood using G-DEXTMIIb Genomic DNA Extraction Kit (iNtRoN Biotechnology, Korea) and pair-end reads were generated from the isolated DNA using Illumina HiSeq 2000. The Covaris System was used to shear 3 μg of genomic DNA into the ~300 bp inserts. The fragments of the sheared DNA were end-repaired, polyA-tailed, adaptor ligated, and amplified using the TruSeq DNA Sample Prep. Kit (Illumina, USA). Pair-end sequencing was performed on the Illumina HiSeq 2000 platform using the TruSeq SBS Kit v3-HS (Illumina, USA) (https://www.illumina.com/documents/products/datasheets/datasheet_hiseq2000.pdf). Finally, sequence data were generated using the Illumina HiSeq system. The details of data are described in [65, 66].

The quality check was carried out on the 6.50 billion reads (~644 Gbp), derived from the genomes of five indigenous African cattle (Ankole, Boran, Kenana, N’Dama and Ogaden) and four commercial cattle breeds (Angus, Jersey, Holstein and Hanwoo), via the fastQC package (http://www.bioinformatics.babraham.ac.uk/projects/fastqc). The pair-end sequence reads were aligned to the UMD 3.1 using Bowtie [67] with the default parameters (except the “-no-mixed” option). The UMD 3.1 reference genome (ftp://ftp.ensembl.org/pub/release-75/fasta/bos_taurus/) from the Ensembl database (release 75) was used as the bovine reference genome for the assembly. The size of reference genome sequence UMD 3.1 is 2.67Gb. The overall alignment rate of the reads to the reference genome was 98.84% with an average read depth of ~10.8X genome coverage. On average across the whole samples, the reads covered 98.56% of the reference UMD3.1 genome (Additional file 1: Table S1).

We used Picard (http://broadinstitute.github.io/picard/) and SAMtools [68] for downstream processing and variant calling. Potential PCR duplicates were filtered using Picard (“REMOVE_DUPLICATEDS = true” option in “MarkDuplicates”), and the index files for the reference and bam files were generated with SAMtools. We also conducted a local multiple sequence realignment to correct misalignments caused by the presence of INDELs (“RealignerTargetCreator” and “IndelRealigner”) and called candidate SNPs (“UnifiedGenotyper” and “SelectVariants”) using GATK 3.1 [69]. After the variants were called and exported into the variant call format (VCF), we filtered the variants to minimize the false positives (“VariantFiltration”). The variants were filtered with the following options: QUAL (Phred-scaled quality score) < 30; MQ0 (the number of reads with a mapping quality of zero) > 4; QD (variants confidence/quality by depth) < 5; and FS (Phred-scaled *p*-value using Fisher’s exact test) > 200. BEAGLE [70] was used to impute missing genotypes and infer haplotype phases. Finally, we obtained ~ 37 million SNPs (Additional file 1: Table S2).

We additionally genotyped 45 African cattle samples (of which blood samples were available) using BovineSNP50 Genotyping BeadChip (Illumina, USA). After filtering out SNPs based on GeneCall score less than 0.7, common loci of SNP chip and DNA resequencing data were extracted and examined to assess concordance (Additional file 1: Table S3).

Moreover, we performed enrichment analysis to detect significant breed-specific SNPs using SNPSift for focusing on the non-synonymous SNPs (MISSENSE and NONSENSE) [71]. SNPSift CaseControl counts the number of genotypes present in two factors, and then a *p-*value calculation is calculated using Fisher exact and Cochran-Armitage trend tests. In general, one of the factors is fixed as genetic models which can be dominant, recessive, or co-dominant. The other is breed information which was applied in this study for identifying breed-specific enriched SNPs. As a result, we constructed 2 by 2 (dominant or recessive coding / breed-specific group information, specific breed, N’Dama, *versus* the others) or 2 by 3 (co-dominant coding / breed-specific group information) contingency tables, and performed Fisher exact and Cochran-Armitage trend tests for the 2 by 2 and 2 by 3 contingency tables, respectively. A total of 37,363,436 SNPs were applied in the tests, and we used Bonferroni correction for multiple correction testing. After identifying significant N’Dama breed-specific enriched SNPs, we annotated each SNP using snpEff (Table 1 and Additional file 1: Table S10).

### Data representation

For effectively representing breed-specific SNP variations, all SNP alleles of the samples are converted into binary values including 0 and 1. ‘0’ denotes the major allele of a SNP position for all the samples while ‘1’ represents minor values regardless of its alleles. This biallelic representation explicitly characterizes the ratio of major and minor alleles of each SNP position per breed, thus allowing breed-specific SNPs to be effectively discovered. To be specific, the allele of the *i*-th SNP is transformed as follows:

$$ S N{P}_i=\left\{\begin{array}{c}\hfill 0,\kern0.48em \mathrm{if}\  SN{P}_i^{*}= Major(i)\hfill \\ {}\hfill 1,\ \mathrm{Otherwise}\kern3.75em \hfill \end{array}\right. $$,

where *SNP*_i_^*^ and *Major*(*i*) are the *i*-th SNP allele and the most frequent allele in the *i*-th SNP position for all the cattle samples. The values 0 and 1 in a SNP position per each breed denote “conserved” and “mutated”, respectively.

### Mutual information analysis

Information-theoretic measures have emerged as a useful way to quantify the dependencies of many genetic variables [72]. In particular, mutual information (MI) of two random variables is an entropy-based metric for measuring mutual dependency between the variables [73]. Several studies using the MI method exist to analyze biological phenomena, however, most of them have been applied to gene expression data [74–78]. This study proposed a hybrid approach based on MI combining statistical methods to detect breed-specific SNPs from large-scale genome sequences.

In genetic association studies, MI can be used to measure the dependencies between genetic factors and phenotypes by defining genetic features and phenotypic classes as random variables. Extracting the discriminative genetic variations from tens of millions of SNPs can be addressed as finding the distinct variables from a huge-scale variable set. Given a SNP position variable set *X* = {*x*_*1*_,…,*x*_*n*_} and a breed class variable *y*, we define a function *F*(*X*;*y*) that selects variables by measuring the associations between SNP positions and breed classes:

$$ {X}^{*}= F\left( X; y\right)={\displaystyle \underset{i, j}{\cup}\kern0.5em  f\left({x}_i,{x}_j, y\right)} $$

$$ \mathrm{s}.\mathrm{t}.\kern0.5em  f\left({x}_i,{x}_j, y\right)=\left\{\begin{array}{c}\hfill \left\{\left({x}_i,{x}_j\right)\right\},\mathrm{if}\kern0.5em  MIE\left({x}_i,{x}_j, y\right)>\theta \hfill \\ {}\hfill \varnothing, \kern4em \mathrm{otherwise}\hfill \end{array}\right. $$

where *X* ^*^ is the selective SNP variable pair set, *x*_*i*_ and *x*_*j*_ denote two SNP variables in a chromosome, and *θ* indicates the threshold for selecting the SNP variables. Also, MIE denotes mutual information estimator.

When the two random variables, *SNP* and *C*, denotes a genetic variable and phenotypic class, respectively, the value set of a *SNP* consists of its possible alleles, and the value set of *C* is defined as {N’Dama, other cattle}. The MI *I*(*SNP*; *C*) quantifies the reduction in the uncertainty of the phenotypic class *C* due to the information contained in the genetic variation of *SNP*:

$$ I\left( SNP; C\right)= H(SNP)- H\left( SNP\left| C\right.\right) $$

$$ \mathrm{s}.\mathrm{t}.\kern0.5em  H(SNP)=-{\displaystyle {\sum}_{snp\in SNP} p(snp) \log \kern0.5em  p(snp),\kern0.5em \mathrm{and}\kern0.5em  H\left( SNP\left| C\right.\right)- H(C).} $$

where *H*(*SNP*) is the entropy of *SNP. H*(*SNP*|*C*) denotes the conditional entropy of *SNP* for a given *C,* and it can be found using the chain rule. Thus, by the definition of the entropy *H*, the MI can be reformulated with the joint probability distribution *p*(*SNP*, *C*) as follows:

$$ I\left( SNP; C\right)={\displaystyle \sum_{snp\in SNP}{\displaystyle \sum_{c\in C} p\left( snp, c\right)\kern0.5em  \log \kern0.5em \frac{p\left( snp, c\right)}{p\left( snp, c\right)\kern0.5em  p(c)}}} $$

*I*(*SNP*; *C*) is nonnegative and is only zero when *p*(*SNP*, *C*) = *p*(*SNP*)*p*(*C*), indicating that there is no association between *SNP* and *C*. Intuitively, then, MIEs can be used for measuring the main effect of a genetic variable *SNP* on the breed *C*.

In this study, we calculated conditional mutual information (conditional MI) to quantify the associations among three or more variables as the MIE function and to measure the influence of two-locus haplotypes on the breeds. Conditional MI is defined as follows:

$$ I\left( C; SN{P}_1\left| SN{P}_2\right.\right)= I\left( C; SN{P}_1, SNP\right)- I\left( C; SN{P}_2\right). $$

We defined *I*(*C*; *SNP*_*1*_, *SNP*_*2*_) as an MIE. MIEs can be obtained by the chain rule for MI:

$$ I\left( C; SN{P}_1, SN{P}_2\right)= I\left( C; SN{P}_1\left| SN{P}_2\right.\right)+ I\left( C; SN{P}_2\right), $$

$$ \mathrm{s}.\mathrm{t}.\kern0.5em  I\left( C; SN{P}_1\left| SN{P}_2\right.\right)={\displaystyle \sum_{s_2\in SN{P}_2}{\displaystyle \sum_{s_1\in SN{P}_1}{\displaystyle \sum_{c\in C}{P}_{C, SN{P}_1, SN{P}_2}\left( c,{s}_1,{s}_2\right) \log \kern0.5em \frac{p_{SN{P}_2}\left({s}_2\right){p}_{C, SN{P}_1, SN{P}_2}\left( c,{s}_1,{s}_2\right)}{{p_{C,}}_{SN{P}_2}\left( c,{s}_2\right){p}_{SN{P}_1, SN{P}_2}\left({s}_1,{s}_2\right)}}}} $$

The MIE quantifies the associations between SNPs at two loci and breeds. *I*(*C*; *SNP*_*1*_ | *SNP*_*2*_) is also nonnegative and becomes zero when no dependency exists among all three variables. This property allows the method to be suitable for identifying distinct two-locus haplotypes determining the phenotype of cattle.

Weighted MI between the *i*-th gene and breed variable *C* (wMI) is defined by interpolating the number of SNPs annotated by the gene and the mean MI of the gene:

$$ w M{I}_i=\alpha \overline{I}\left({g}_i; C\right)+\left(1-\alpha \right)\frac{\left|{g}_i\right|}{\underset{g\in G}{ \max}\left\{\left| g\right|\right\}} $$

$$ \overline{I}\left({g}_i; C\right)=\frac{1}{\left|{g}_i\right|}{\displaystyle {\sum}_{SNP\in {g}_i} I\left( SNP; C\right)} $$

where *g*_*i*_ is the set of SNPs annotated by the *i*-th gene and *α* denotes the constant for moderating two factors. When a gene possesses more SNPs and mean MI between the gene SNPs and the breed variable is larger, the wMI of the gene provides a larger value.

### XP-CLR and XP-EHH tests

We performed cross-population composite likelihood ratio (XP-CLR) and cross-population extended haplotype homozygosity (XP-EHH) tests for detecting the selective pressures in the N’Dama and Ogaden cattle. The XP-CLR scores are computed using XP-CLR 1.0 (https://reich.hms.harvard.edu/software) for observation of selective sweeps which involve modeling the multi-locus allele frequency differentiation between two populations [21, 49]. The parameters including non-overlapping sliding windows of 50 kb, a maximum number of SNPs within each window of 600, and the correlation level of the SNPs’ contribution to the XP-CLR results down-weighted of 0.95 are used. The regions with XP-CLR scores in the top 1% of the empirical distributions (XP-CLR > 224.2) are designated as candidate sweeps in the N’Dama and Ogaden breeds (Additional file 1: Table S12).

In addition, we used the XP-EHH to identify the loci of selection based on the comparison of genome-wide SNP genotypes between populations. The XP-EHH scores are calculated using software xpehh (http://hgdp.uchicago.edu/Software/) to detect alleles with an increase in frequency to the point of fixation or near-fixation in one of the populations. It means that it detects SNPs which are under selection in one population but not in others. So, the extreme XP-EHH scores potentially represent the selection of a particular population. XP-EHH scores are also directional. A positive score means that selection is likely to have happened in population A, while a negative score indicates the selection probably occurs in population B [21, 79]. The genome is divided into non-overlapping segments of 50 kb to facilitate the comparison of genomic regions across populations, before calculation of the maximum XP-EHH score of all SNPs in each segment. We binned genomic windows according to their numbers of SNPs in the increments of 500 SNPs to consider the SNP frequency. Within each bin, for each window *i*, the fraction of windows with a value of the statistic greater than that in *i* is defined as the empirical *p*-value [21, 80]. The resulting XP-EHH value with a positive score indicates selection in the N’Dama, whereas a negative score signifies selection in the Ogaden.

We selected the regions with positive XP-EHH scores in *p*-values less than 1%, which can be considered as strong signals in the N’Dama breed (Additional file 1: Table S13). Finally, the selected genomic regions found from XP-CLR and XP-EHH tests are annotated to the closest genes (UMD 3.1). Genes that partially or completely span the window regions (-25 ~ + 25 kb) are defined as candidate genes.

### Construction of gene interaction networks based on genetic variations between the breeds

A gene correlation network characterizes the correlation of the variations of genes for cattle breeds. The patterns of genetic variations based on the converted SNP alleles which are distinguishable from cattle breeds are used to build the gene-gene interaction networks. The networks constructed from the annotated genes and their quantitative variation degrees in a gene level are as follows:

1. We select the genes with a significant level of *p*-value < 1.0e-3 with respect to wMI, the intersection of MI and XP-CLR, and of MI and XP-EHH.
2. An allele pair for the selected SNPs was converted into a three-level value with respect to variation status as 0, 1 and 2 by summing the pair. The converted SNP values are 0 or 2 when a SNP allele pair in a position is major homozygous types or alternative homozygous types, respectively. When an allele pair shows heterozygosity, on the other hands, the value is 1. For example, we assume that the alleles of a SNP position pair belongs to “AA”, “AT”, or “TT”, and “A” is a major allele of the SNP pair. Then, the SNP value in this position for all samples are converted into 0, 1 or 2, respectively. This value is defined as the SNP variations of each sample.
3. The selected SNPs are annotated by genes in which these SNPs are located.
4. We compute the mean of the SNP values calculated in (2) for each gene. Note that this mean value is defined as the variation of a gene.
5. We calculate the Pearson correlation coefficient of all the gene pairs from the gene variations of cattle breed samples computed in (4):
  $$ Corr\left({g}_i,{g}_j\right)=\frac{Cov\left({g}_i,{g}_j\right)}{\sigma_i{\sigma}_j} $$
  where *g*_*i*_ and *g*_*j*_ denote the *i*-th and the *j*-th gene variations. *σ*_*i*_ and *Cov*(*g*_*i*_, *g*_*j*_) mean the standard deviation of *g*_*i*_ and the covariance of *g*_*i*_ and *g*_*j*_.
6. A gene corresponds to a node and two genes with a significant correlation coefficient are connected to each other.

For investigating N’Dama-specific traits including trypanotolerance, the gene interaction networks are constructed from N’Dama and Ogaden breeds. Also, the positive and the negative thresholds are selected for connecting two genes. We implemented the source code for ourselves using the scipy package of Python 2.7 in order to calculate correlation coefficients between the extracted genes, and used Cytoscape 3.2.1 for network visualization.

Finally, we conduct functional analysis for the genes of the constructed networks using the Database for Annotation, Visualization and Integrated Discovery (DAVID) ver. 6.7 (https://david-d.ncifcrf.gov/tools.jsp) [81] to statistically determine over-representation of GO categories. Go analysis were carried out with default parameters in DAVID which were set to GO level “all”, count threshold (the minimum number of gene for the corresponding GO term) of 2 and EASE threshold of 0.1. EASE score is the modified Fisher exact *p*-value adjustment than the naïve Fisher exact test [82]. We also used FDR to correct the multiple testing errors.

## Additional file

Additional file 1: Supplementary figures and tables. (PDF 2201 kb)

## Abbreviations

ADSCs: Adipose-derived mesenchymal stem cells
FDR: False discovery rate
GO: Gene ontology
GWAS: Genome-wide association study
HSCs: Haematopoietic stem cells
KL-divergence: Kullback-Liebler-divergence
MI: Mutual information
miR: microRNA
SNP: Single-nucleotide polymorphism
VCF: Variant call format
wMI: Weighted mutual information
WNT/PCP: WNT/planar cell polarity
XP-CLR: Cross-population composite likelihood ratio
XP-EHH: Cross-population extended haplotype homozygosity
