# Supplementary material for: Cattle genome-wide analysis reveals genetic signatures in trypanotolerant N’Dama
Source: BMC Genomics. 2017 May 12;18:371. doi: 10.1186/s12864-017-3742-2 (PMC5427609; doi:10.1186/s12864-017-3742-2)
Supplement: Additional file 1: — Supplementary figures and tables. (PDF 2201 kb) [file 12864_2017_3742_MOESM1_ESM.pdf]

Cattle genome-wide analysis reveals genetic signatures in trypanotolerant N'Dama

Soo-Jin Kim, Sojeong Ka, Jung-Woo Ha, Jaemin Kim, DongAhn Yoo, Kwondo Kim, Hak-Kyo Lee, Dajeong Lim, Seoae Cho, Olivier Hanotte, Okeyo Ally Mwai, Tadelle Dessie, Stephen Kemp, Sung Jong Oh*, Heebal Kim*

SUPPLEMENTARY INFORMATION

Supplementary Tables **2-29**

Table S1 2

Table S2 5

Table S3 6

Table S4 8

Table S5 9

Table S6 10

Table S7 14

Table S8 15

Table S9 19

Table S10 20

Table S11 21

Table S12 22

Table S13 26

Supplementary Figures **30-34**

Figure S1 30

Figure S2 31

Figure S3 32

Figure S4 33

Figure S5 34

References **35-36**

Table S1. Summary of African cattle and commercial cattle sequencing data.

Sample ID	DNA sequenced (bp)	Total reads	Alignment rate (%)	Read depth	Genome coverage (%)
Ankole_01	30,125,956,396	298,276,796	98.76	9.63	98.66
Ankole_02	26,850,027,254	265,841,854	98.91	8.81	98.60
Ankole_03	28,511,065,982	282,287,782	89.16	6.92	95.31
Ankole_04	31,302,879,904	309,929,504	98.99	9.91	98.67
Ankole_05	30,310,459,964	300,103,564	98.89	9.82	98.73
Ankole_06	30,781,515,884	304,767,484	98.85	10.04	98.61
Ankole_07	30,329,107,796	300,288,196	99.01	9.89	98.61
Ankole_08	29,454,188,226	291,625,626	98.96	9.47	98.64
Ankole_09	28,797,582,580	285,124,580	98.94	9.42	98.64
Ankole_10	29,320,591,284	290,302,884	98.96	9.43	98.65
Boran_01	30,638,401,106	303,350,506	98.77	5.97	97.78
Boran_02	28,403,655,310	281,224,310	98.92	9.07	98.61
Boran_03	28,301,084,356	280,208,756	98.64	9.09	98.56
Boran_04	28,325,968,534	280,455,134	98.58	8.99	98.56
Boran_05	31,211,002,224	309,019,824	98.90	9.98	98.66
Boran_06	30,304,683,976	300,046,376	98.77	9.51	98.61
Boran_07	28,267,720,016	279,878,416	98.76	8.87	98.53
Boran_08	31,921,906,076	316,058,476	98.64	9.67	98.68
Boran_09	28,829,275,168	285,438,368	98.80	9.15	98.55
Boran_10	32,477,775,534	321,562,134	96.16	9.85	98.73
Kenana_01	30,839,778,340	305,344,340	98.87	9.69	98.61
Kenana_02	31,233,638,748	309,243,948	98.80	9.80	98.63
Kenana_03	31,017,693,678	307,105,878	98.78	9.74	98.59
Kenana_04	30,171,253,078	298,725,278	98.78	9.45	98.60
Kenana_05	30,161,939,666	298,633,066	98.79	9.67	98.60
Kenana_06	29,802,497,432	295,074,232	98.84	9.52	98.61
Kenana_07	30,075,057,244	297,772,844	98.78	9.52	98.59
Kenana_08	30,979,082,186	306,723,586	98.74	9.63	98.74
Kenana_09	30,609,308,258	303,062,458	98.87	9.68	98.61
N’Dama_01	30,210,384,316	299,112,716	98.99	9.96	98.65
N’Dama_02	28,909,114,052	286,228,852	99.09	9.31	98.62
N’Dama_03	27,437,854,324	271,661,924	98.11	8.73	98.56
N’Dama_04	30,088,935,654	297,910,254	99.09	9.72	98.63
N’Dama_05	30,552,295,374	302,497,974	99.08	9.96	98.66
N’Dama_06	30,441,334,552	301,399,352	99.14	10.20	98.70
N’Dama_07	30,187,321,774	298,884,374	99.12	9.79	98.66
N’Dama_08	29,920,318,376	296,240,776	98.96	9.48	98.65

N’Dama_09	31,590,149,558	312,773,758	99.12	10.07	98.67
N’Dama_10	30,095,727,500	297,977,500	99.10	9.76	98.65
Ogaden_01	29,246,322,550	289,567,550	98.60	8.92	98.54
Ogaden_02	31,562,494,748	312,499,948	98.74	9.95	98.61
Ogaden_03	29,799,017,376	295,039,776	98.72	9.40	98.57
Ogaden_04	29,461,012,594	291,693,194	98.68	9.28	98.58
Ogaden_05	30,480,606,584	301,788,184	98.71	9.63	98.61
Ogaden_06	31,422,123,736	311,110,136	98.79	9.41	98.55
Ogaden_07	29,744,012,574	294,495,174	98.54	9.14	98.54
Ogaden_08	26,868,959,300	266,029,300	98.49	8.58	98.66
Ogaden_09	31,218,873,356	309,097,756	98.62	9.67	98.69
Angus_01	38,297,780,344	379,185,944	98.92	11.64	98.92
Angus_02	42,897,945,736	424,732,136	98.96	12.55	98.77
Angus_03	42,235,455,224	418,172,824	98.95	12.48	98.91
Angus_04	42,375,411,328	419,558,528	98.96	12.65	98.87
Angus_05	41,996,691,628	415,808,828	99.00	12.59	98.74
Angus_06	41,321,509,052	409,123,852	99.13	12.22	98.84
Angus_07	39,340,897,840	389,513,840	99.24	12.15	98.71
Angus_08	38,829,290,824	384,448,424	99.22	12.13	98.66
Angus_09	41,473,927,142	410,632,942	99.10	9.75	98.88
Angus_10	42,082,054,808	416,654,008	99.19	8.28	98.57
Jersey_01	47,465,593,168	469,956,368	99.07	15.36	98.76
Jersey_02	52,351,160,118	518,328,318	99.29	16.92	98.62
Jersey_03	45,008,930,980	445,632,980	99.36	13.18	98.54
Jersey_04	45,163,648,638	447,164,838	99.29	13.58	98.72
Jersey_05	42,638,456,738	422,162,938	99.36	12.54	98.52
Jersey_06	48,780,958,588	482,979,788	99.38	14.92	98.58
Jersey_07	48,475,329,356	479,953,756	99.31	15.35	98.76
Jersey_08	50,467,011,582	499,673,382	99.34	16.44	98.63
Jersey_09	54,928,496,096	543,846,496	99.13	14.16	98.75
Jersey_10	48,297,886,294	478,196,894	99.32	16.11	98.62
Holstein_01	34,443,272,298	341,022,498	99.18	9.71	98.26
Holstein_02	49,060,911,398	485,751,598	99.12	9.73	98.25
Holstein_03	48,609,505,836	481,282,236	99.18	11.27	98.16
Holstein_04	43,631,921,220	431,999,220	99.08	9.05	98.30
Holstein_05	49,499,785,082	490,096,882	98.98	11.99	98.49
Holstein_06	45,562,688,326	451,115,726	99.17	12.45	98.47
Holstein_07	48,351,766,764	478,730,364	99.13	12.63	98.43
Holstein_08	48,434,849,970	479,552,970	98.90	11.53	98.57

Holstein_09	48,247,230,552	477,695,352	95.67	11.64	98.33
Holstein_10	46,523,896,640	460,632,640	99.17	12.72	98.46
Hanwoo_01	41,335,663,192	409,263,992	99.12	12.87	98.75
Hanwoo_02	44,100,196,004	436,635,604	99.08	13.42	98.83
Hanwoo_03	41,021,933,356	406,157,756	99.07	12.66	98.72
Hanwoo_04	43,342,534,000	429,134,000	99.10	13.17	98.81
Hanwoo_05	39,797,424,708	394,033,908	99.11	12.43	98.79
Hanwoo_06	43,664,654,108	432,323,308	99.13	13.45	98.80
Hanwoo_07	43,179,873,298	427,523,498	99.12	13.34	98.84
Hanwoo_08	38,923,035,388	385,376,588	98.42	12.11	98.73
Hanwoo_09	39,770,122,186	393,763,586	99.09	12.32	98.77
Hanwoo_10	35,730,306,612	353,765,412	99.05	11.27	98.75
Hanwoo_11	35,605,606,356	352,530,756	99.09	11.21	98.71
Hanwoo_12	37,069,123,222	367,021,022	99.08	11.55	98.79
Hanwoo_13	47,383,111,720	469,139,720	99.28	10.49	98.19
Hanwoo_14	37,825,177,710	374,506,710	99.26	8.09	98.28
Hanwoo_15	48,377,581,152	478,985,952	99.27	12.29	98.04
Hanwoo_16	47,887,156,866	474,130,266	99.28	10.08	98.45
Hanwoo_17	44,021,250,768	435,853,968	99.26	11.43	98.13
Hanwoo_18	41,150,976,410	407,435,410	99.17	10.02	98.46
Hanwoo_19	44,308,415,180	438,697,180	99.28	10.82	98.15
Hanwoo_20	42,931,233,922	425,061,722	99.25	13.24	98.55
Hanwoo_21	49,788,867,282	492,959,082	98.94	10.82	98.49
Hanwoo_22	47,111,708,156	466,452,556	99.25	10.44	98.52
Hanwoo_23	43,957,650,260	435,224,260	99.25	9.33	98.41

Table S2. Chromosomal distribution and number of SNPs

Chromosome	Length (bp)*	Number of SNPs	Avg. distance between SNPs
**1**	158,337,067	2,307,435	68
**2**	137,060,424	1,917,262	71
**3**	121,430,405	1,662,248	73
**4**	120,829,699	1,762,569	68
**5**	121,191,424	1,665,070	72
**6**	119,458,736	1,740,133	68
**7**	112,638,659	1,533,299	73
**8**	113,384,836	1,579,856	71
**9**	105,708,250	1,492,154	70
**10**	104,305,016	1,476,536	70
**11**	107,310,763	1,506,893	71
**12**	91,163,125	1,423,996	64
**13**	84,240,350	1,158,798	72
**14**	84,648,390	1,187,482	71
**15**	85,296,676	1,291,360	66
**16**	81,724,687	1,167,512	69
**17**	75,158,596	1,110,369	67
**18**	66,004,023	907,819	72
**19**	64,057,457	875,206	73
**20**	72,042,655	1,068,977	67
**21**	71,599,096	1,031,652	69
**22**	61,435,874	863,499	71
**23**	52,530,062	864,636	60
**24**	62,714,930	948,245	66
**25**	42,904,170	627,563	68
**26**	51,681,464	767,090	67
**27**	45,407,902	719,364	63
**28**	46,312,546	742,945	62
**29**	51,505,224	819,853	62

*Based on UMD3.1 genome of reference

Table S3. Genotype concordance rates of African cattle samples

Sample ID	Loci in common	Concordant loci by genotype	Genotype concordance (%)
Ankole_01	34,091	31,413	92.14
Ankole_02	36,590	34,834	95.20
Ankole_03	36,802	32,869	89.31
Ankole_04	36,215	34,600	95.54
Ankole_05	33,673	29,075	86.35
Ankole_06	30,323	28,911	95.34
Ankole_07	36,084	34,508	95.63
Ankole_08	36,899	35,252	95.54
Ankole_09	36,647	34,229	93.40
Ankole_10	35,651	32,120	90.10
Boran_01	36,403	34,656	95.20
Boran_02	36,321	34,461	94.88
Boran_03	36,153	34,344	95.00
Boran_04	30,075	28,268	93.99
Boran_05	26,829	25,200	93.93
Boran_07	33,544	31,553	94.06
Boran_08	30,109	28,597	94.98
Boran_09	35,556	33,858	95.22
Kenana_01	40,392	38,872	96.24
Kenana_02	40,001	38,463	96.16
Kenana_03	35,577	34,148	95.98
Kenana_04	35,154	33,746	95.99
Kenana_06	33,527	31,940	95.27
Kenana_07	34,716	33,289	95.89
Kenana_08	37,535	35,941	95.75
Kenana_09	39,093	37,601	96.18
N’Dama_01	37,028	35,506	95.89
N’Dama_02	36,904	35,210	95.41
N’Dama_03	36,327	34,249	94.28
N’Dama_04	32,033	30,241	94.41
N’Dama_05	29,075	27,532	94.69
N’Dama_06	39,324	37,798	96.12
N’Dama_07	41,238	39,432	95.62
N’Dama_08	40,885	39,209	95.90
N’Dama_09	38,286	36,923	96.44
N’Dama_10	38,164	36,812	96.46
Ogaden_01	39,454	37,828	95.88
Ogaden_02	40,108	38,557	96.13

Ogaden_03	36,741	35,075	95.47
Ogaden_04	34,983	33,428	95.55
Ogaden_05	35,423	33,944	95.82
Ogaden_06	38,686	37,104	95.91
Ogaden_07	39,525	37,982	96.10
Ogaden_08	39,689	38,051	95.87
Ogaden_09	36,827	35,420	96.18

Table S4. 30 identified genes based on weighted MI (wMI) and the number of the identified SNPs.

Ensemble Gene ID	Gene Symbol	Description	Location	#SNP
ENSBTAG00000044111	EPHA6	EPH Receptor A6	1:40608729-41623778	2
ENSBTAG00000044055	THSD7B	thrombospondin, type I, domain containing 7B	2:59750109-60547022	13
ENSBTAG00000031358	DPYD	dihydropyrimidine dehydrogenase	3:45563732-46487165	53
ENSBTAG00000021905	DGKB	diacylglycerol kinase, beta 90kDa	4:22333433-23127587	467
ENSBTAG00000004398	IMMP2L	inner mitochondrial membrane peptidase subunit 2	4:57127737-58083528	5
ENSBTAG00000014112	EXOC4	exocyst complex component 4	4:97791626-98594890	51
ENSBTAG00000005711	NSG1	Neuron specific gene family member 1	6:106483716-107356158	9
ENSBTAG00000019808	CCSER1	coiled-coil serine-rich protein 1	6:35100701-35938394	113
ENSBTAG00000047743	KCNIP4	Kv channel interacting protein 4	6:41709362-43021626	381
ENSBTAG00000035007	GALNTL6	polypeptide N-acetylgalactosaminyltransferase-like 6	8:3816704-5330369	52
ENSBTAG00000018996	PARK2	E3 ubiquitin-protein ligase parkin	9:98421510-99568077	18
ENSBTAG00000000432	TRAC	T cell receptor alpha constant	10:22106909-23304334	73
ENSBTAG00000031669	CTNNA2	Catenin (Cadherin-Associated Protein), Alpha 2	11:54723190-55906462	110
ENSBTAG00000008708	GPC5	glypican 5	12:66292489-67324791	620
ENSBTAG00000008338	PLCB1	phospholipase C, beta 1 (phosphoinositide-specific)	13:789380-1695139	43
ENSBTAG00000006188	USH2A	Usher syndrome 2A (autosomal recessive, mild)	16:19573856-20502175	51
ENSBTAG00000033180	SMYD3	SET and MYND domain containing 3	16:31589396-32333109	213
ENSBTAG00000015894	WWOX	WW domain containing oxidoreductas	18:5283909-6196774	19
ENSBTAG00000025200	ACCN1	amiloride-sensitive cation channel 1, neuronal, with a synonym of ASIC2	19:16353233-17562209	43
ENSBTAG00000034681	CA10	carbonic anhydrase X	19:627558-1472951	47
ENSBTAG00000013047	GRM7	metabotropic glutamate receptor 7 precursor	22:18740484-19647747	7
ENSBTAG00000044062	RBMS3	RNA binding motif, single stranded interacting protein 3	22:3899285-4695113	39
ENSBTAG00000021911	PTPRG	protein tyrosine phosphatase, receptor type, G	22:39175038-40360572	27
ENSBTAG00000014418	FHIT	fragile histidine triad	22:41319551-42108622	319
ENSBTAG00000012039	PHACTR1	phosphatase and actin regulator 1	23:43052892-43647528	294
ENSBTAG00000045905	PCDH15	protocadherin-related 15	26:5017714-5578654	27
ENSBTAG00000018404	PRKG1	protein kinase, cGMP-dependent, type I	26:6906081-8343629	678
ENSBTAG00000045699	CTNNA3	catenin (cadherin-associated protein), alpha 3	28:22419203-24270401	3
ENSBTAG00000004081	FAT3	FAT atypical cadherin 3	29:1965869-2605125	103
ENSBTAG0000004xxxx	U6	U6 spliceosomal RNA	multiple loci	6879

Table S5. Significant GO terms in biological process from the extracted genes of the correlation network constructed based on the weighted MI (*P*-value less than 0.05).

GO:ID	GO terms	Associated genes	*P*-value*	FDR
**GO:0050877**	neurological system process	ACCN1, GRM7, PCDH15, PARK2, PLCB1, USH2A, CTNNA2	3.4E-05	4.2E-04
**GO:0007612**	learning	GRM7, PARK2, PLCB1	6.8E-04	8.5E-03
**GO:0050905**	neuromuscular process	PCDH15, PARK2, CTNNA2	7.3E-04	9.1E-03
**GO:0007268**	synaptic transmission	ACCN1, GRM7, PARK2, CTNNA2	8.1E-04	1.0E-02
**GO:0019226**	transmission of nerve impulse	ACCN1, GRM7, PARK2, CTNNA2	1.3E-03	1.6E-02
**GO:0007605**	sensory perception of sound	GRM7, PCDH15, USH2A	1.8E-03	2.2E-02
**GO:0050890**	cognition	GRM7, PCDH15, PARK2, PLCB1, USH2A	1.9E-03	2.4E-02
**GO:0050954**	sensory perception of mechanical stimulus	GRM7, PCDH15, USH2A	2.0E-03	2.5E-02
**GO:0007611**	learning or memory	GRM7, PARK2, PLCB1	2.3E-03	2.9E-02
**GO:0007267**	cell-cell signaling	ACCN1, GRM7, PARK2, CTNNA2, FHIT	6.0E-03	7.2E-02
**GO:0001964**	startle response	PARK2, CTNNA2	1.1E-02	1.2E-01
**GO:0045494**	photoreceptor cell maintenance	PCDH15, USH2A	1.4E-02	1.6E-01
**GO:0043954**	cellular component maintenance	PCDH15, USH2A, FHIT	1.7E-02	1.9E-01
**GO:0007613**	memory	GRM7, PLCB1	2.6E-02	2.8E-01
**GO:0007610**	behavior	GRM7, PARK2, PLCB1	3.7E-02	3.7E-01

* *P*-value is the modified Fisher exact *p*-value.

Table S6. 131 identified genes based on MI and XP-CLR and the number of the identified SNPs.

Ensemble Gene ID	Gene Symbol	Description	Location	#SNP
ENSBTAG00000009942	PLCL2	Bos taurus phospholipase C-like 2 (PLCL2), mRNA.	1:155618262-155833445	32
ENSBTAG00000013215	MREG	Bos taurus melanoregulin (MREG), mRNA.	2:104492355-104560643	80
ENSBTAG00000016819	FABP3	Bos taurus fatty acid binding protein 3, muscle and heart (mammary-derived growth inhibitor) (FABP3), mRNA.	2:122723225-122783830	7
ENSBTAG00000013309	SFRS4	serine/arginine-rich splicing factor 4	2:124956594-124982238	26
ENSBTAG00000020769	OPCML	opioid binding protein/cell adhesion molecule-like	2:125010196-125011119	44
ENSBTAG00000047524	TMEM200B	transmembrane protein 200B	2:125010196-125011119	56
ENSBTAG00000006667	EPB41	Bos taurus erythrocyte membrane protein band 4.1 (elliptocytosis 1, RH-linked)	2:125014818-125113212	139
ENSBTAG00000008766	SLC9A1	Bos taurus solute carrier family 9 (sodium/hydrogen exchanger), member 1 (SLC9A1), mRNA.	2:126676550-126727482	31
ENSBTAG00000013048	NIPAL3	NIPA-like domain containing 3	2:129041413-129091075	114
ENSBTAG00000010498	SLC40A1	Bos taurus solute carrier family 40 (iron-regulated transporter), member 1 (SLC40A1), mRNA.	2:6716591-6740329	5
ENSBTAG00000003002	WDR75	WD repeat domain 75	2:6875268-6916093	180
ENSBTAG00000003217	CADM3	Bos taurus cell adhesion molecule 3 (CADM3), mRNA.	3:10652468-10683411	68
ENSBTAG00000047314	PPIE	Bos taurus peptidylprolyl isomerase E (cyclophilin E) (PPIE), mRNA.	3:106917951-106934190	49
ENSBTAG00000019555	ZSCAN20	zinc finger and SCAN domain containing 20	3:112931814-112942579	7
ENSBTAG00000026181	UGT1A6	Bos taurus UDP glucuronosyltransferase 1 family, polypeptide A6, mRNA.	3:113907720-114031371	215
ENSBTAG00000013505	IQCA1	Bos taurus IQ motif containing with AAA domain 1 (IQCA1), mRNA.	3:116402594-116592405	92
ENSBTAG00000044150	UCK2	Bos taurus uridine-cytidine kinase 2 (UCK2), mRNA.	3:3045781-3119590	35
ENSBTAG00000017458	CALCR	calcitonin receptor	4:10614458-10724579	186
ENSBTAG00000014372	SMARCD3	SWI/SNF related, matrix associated, actin dependent regulator of chromatin, subfamily d, member 3	4:114615444-114680160	35
ENSBTAG00000006232	WDR86	Bos taurus WD repeat domain 86 (WDR86), mRNA.	4:114730382-114755250	49
ENSBTAG00000003019	CRYGN	crystallin, gamma N	4:114773449-114779653	18
ENSBTAG00000011819	PHF14	PHD finger protein 14	4:18997840-19093243	60
ENSBTAG00000044023	CDK6	cyclin-dependent kinase 6	4:9792303-10039520	14
ENSBTAG00000017378	GALNT8	polypeptide N-acetylgalactosaminyltransferase 8	5:105799704-105837839	197
ENSBTAG00000030343	FGF23	fibroblast growth factor 23	5:106208179-106216757	93
ENSBTAG00000016650	TIGAR	TP53 induced glycolysis regulatory phosphatase	5:106223071-106238040	82
ENSBTAG00000003036	PPFIA2	protein tyrosine phosphatase, receptor type, f polypeptide (PTPRF), interacting protein (liprin), alpha 2	5:10913178-11118179	23
ENSBTAG00000006200	SLC25A17	Bos taurus solute carrier family 25 (mitochondrial carrier; peroxisomal membrane protein, 34kDa), member 17 (SLC25A17), mRNA.	5:112564970-112607538	87

ENSBTAG00000001057	ARFGAP3	Bos taurus ADP-ribosylation factor GTPase activating protein 3, mRNA.	5:114291727-114341661	37
ENSBTAG00000014156	AMN1	antagonist of mitotic exit network 1 homolog (S. cerevisiae)	5:78552287-78637341	17
ENSBTAG00000020715	PIK3C2G	phosphatidylinositol-4-phosphate 3-kinase, catalytic subunit type 2 gamma	5:91835146-92276939	298
ENSBTAG00000006256	PTPRO	protein tyrosine phosphatase, receptor type, O	5:94770904-94881418	95
ENSBTAG00000012343	TSPAN5	Bos taurus tetraspanin 5 (TSPAN5), mRNA.	6:27333479-27512867	160
ENSBTAG00000004653	GPR125	G protein-coupled receptor 125,	6:43436544-43531420	25
ENSBTAG00000019708	ACSL6	Bos taurus acyl-CoA synthetase long-chain family member 6 (ACSL6), mRNA.	7:23779096-23882198	133
ENSBTAG00000005961	CDC42SE2	Bos taurus CDC42 small effector 2 (CDC42SE2), mRNA.	7:24318564-24436629	133
ENSBTAG00000038489	DCDC2C	doublecortin domain containing 2C	8:112796601-112836172	17
ENSBTAG00000013838	ALLC	Bos taurus allantoicase (ALLC), mRNA.	8:112837179-112860706	27
ENSBTAG00000016225	COLEC11	Bos taurus collectin sub-family member 11 (COLEC11), mRNA.	8:112869671-112892968	29
ENSBTAG00000025853	HOMER1	Bos taurus homer homolog 1 (Drosophila) (HOMER1), mRNA.	10:10350692-10484916	100
ENSBTAG00000006440	IGDCC4	immunoglobulin superfamily, DCC subclass, member 4	10:12264594-12291146	64
ENSBTAG00000000218	MAP2K5	Bos taurus mitogen-activated protein kinase kinase 5 (MAP2K5), mRNA.	10:14354690-14626128	62
ENSBTAG00000003043	GNG2	Bos taurus guanine nucleotide binding protein (G protein), gamma 2, mRNA.	10:44711724-44842190	1
ENSBTAG00000027080	SLC8A3	solute carrier family 8 (sodium/calcium exchanger), member 3	10:82128379-82273227	54
ENSBTAG00000007371	SCAMP1	Bos taurus secretory carrier membrane protein 1 (SCAMP1), mRNA.	10:9369310-9520700	13
ENSBTAG00000032521	PLEKHH2	pleckstrin homology domain containing, family H (with MyTH4 domain) member 2	11:25974409-26070971	64
ENSBTAG00000020614	PRKCE	protein kinase C, epsilon	11:27935104-28472632	117
ENSBTAG00000005828	MERTK	Bos taurus c-mer proto-oncogene tyrosine kinase (MERTK), mRNA.	11:332458-427685	33
ENSBTAG00000038011	SH3RF3	SH3 domain containing ring finger 3	11:44206549-44365502	11
ENSBTAG00000008272	EBF3	early B-cell factor 3	11:48848624-48905318	17
ENSBTAG00000011601	ST3GAL5	ST3 beta-galactoside alpha-2,3-sialyltransferase 5	11:48848624-48905318	91
ENSBTAG00000005273	IL1R1	interleukin 1 receptor, type I	11:6876948-6929909	5
ENSBTAG00000005704	IL1RL2	interleukin 1 receptor-like 2	11:7020020-7045388	54
ENSBTAG00000018571	IL1RL1	interleukin 1 receptor-like 1	11:7121609-7141103	110
ENSBTAG00000002271	CDADC1	cytidine and dCMP deaminase domain containing 1	12:18962979-18990944	295
ENSBTAG00000034222	CAB39L	calcium binding protein 39-like	12:19016368-19079171	182
ENSBTAG00000034269	RCBTB1	regulator of chromosome condensation (RCC1) and BTB (POZ) domain containing protein 1	12:19194202-19250906	31
ENSBTAG00000016998	ARL11	Bos taurus ADP-ribosylation factor-like 11 (ARL11), mRNA.	12:19275074-19276925	38
ENSBTAG00000004595	GML	Bos taurus glycosylphosphatidylinositol anchored molecule like protein, mRNA.	14:2715416-2742638	64
ENSBTAG00000016210	LYPD2	LY6/PLAUR domain containing 2	14:2829285-2831334	27
ENSBTAG00000000654	ARMC4	armadillo repeat containing 4	14:2834781-2835985	47
ENSBTAG00000016209	SLURP1	secreted LY6/PLAUR domain containing 1	14:2834781-2835985	25
ENSBTAG00000000158	LY6K	lymphocyte antigen 6 complex, locus K	14:2839180-2842655	59

ENSBTAG00000005063	THEM6	Bos taurus thioesterase superfamily member 6 (THEM6), mRNA.	14:2839180-2842655	22
ENSBTAG00000047022	PSCA	prostate stem cell antigen	14:2854604-2856167	11
ENSBTAG00000006877	MMP-16	matrix metallopeptidase 16 (membrane-inserted)	14:76757695-77111545	101
ENSBTAG00000021796	C12orf43	chromosome 12 open reading frame 43	15:36600420-37082361	13
ENSBTAG00000044185	SOX6	SRY (sex determining region Y)-box 6	15:36600420-37082361	68
ENSBTAG00000008204	C9orf41	chromosome 9 open reading frame 41	15:38991044-39309268	36
ENSBTAG00000009150	SPON1	Bos taurus spondin 1, extracellular matrix protein (SPON1), mRNA.	15:38991044-39309268	216
ENSBTAG00000011578	CD44	Bos taurus CD44 molecule (Indian blood group) (CD44), mRNA.	15:66454331-66541790	80
ENSBTAG00000007019	LRIT3	leucine-rich repeat, immunoglobulin-like and transmembrane domains 3	16:27388710-27522155	12
ENSBTAG00000020989	SUSD4	sushi domain containing 4	16:27388710-27522155	35
ENSBTAG00000022777	CDC42BPA	Bos taurus CDC42 binding protein kinase alpha (DMPK-like), mRNA.	16:30708032-31000733	51
ENSBTAG00000014548	FAM129A	Bos taurus family with sequence similarity 129, member A (FAM129A), mRNA.	16:67318525-67499532	179
ENSBTAG00000013611	SCLT1	sodium channel and clathrin linker 1	17:29190572-29354595	157
ENSBTAG00000001806	IQCD	Bos taurus IQ motif containing D (IQCD), mRNA.	17:63474993-63497850	37
ENSBTAG00000021795	HNF1A	Bos taurus HNF1 homeobox A (HNF1A), mRNA.	17:65417479-65434194	13
ENSBTAG00000003297	OASL	Bos taurus 2'-5'-oligoadenylate synthetase-like (OASL), mRNA.	17:65446987-65463409	13
ENSBTAG00000006608	GGT5	gamma-glutamyltransferase 5	17:73425746-73434932	56
ENSBTAG00000016268	XRCC1	X-ray repair complementing defective repair in Chinese hamster cells 1	18:52081208-52100015	220
ENSBTAG00000005367	VRK3	Bos taurus vaccinia related kinase 3 (VRK3), mRNA.	18:56748766-56784317	45
ENSBTAG00000000803	ZNF667	Bos taurus zinc finger protein 667 (ZNF667), mRNA.	18:63871806-63894090	10
ENSBTAG00000044288	bta-mir-2345	Bos taurus bta-mir-2345	19:50931428-50931490	61
ENSBTAG00000017746	SLIT3	slit homolog 3 protein precursor	20:324518-507045	38
ENSBTAG00000017719	AKAP6	A kinase (PRKA) anchor protein 6	21:43132861-43630707	510
ENSBTAG00000013491	EML1	echinoderm microtubule associated protein like 1, with a synonym of EMAPL	21:66358950-66556801	3
ENSBTAG00000016920	EOMES	eomesodermin	22:2112410-2117440	36
ENSBTAG00000044062	RBMS3	RNA binding motif, single stranded interacting protein 3	22:3899285-4695113	39
ENSBTAG00000001305	ATP2B2	plasma membrane calcium-transporting ATPase 2	22:55001027-55302080	136
ENSBTAG00000003237	IQSEC1	Bos taurus IQ motif and Sec7 domain 1 (IQSEC1), mRNA.	22:59460406-59549010	300
ENSBTAG00000003242	ACAD9	Bos taurus acyl-CoA dehydrogenase family, member 9 (ACAD9), mRNA.	22:59573116-59609270	175
ENSBTAG00000014470	ALDH1L1	Bos taurus aldehyde dehydrogenase 1 family, member L1 (ALDH1L1), mRNA.	22:61221251-61248521	41
ENSBTAG00000004834	CNOT10	Bos taurus CCR4-NOT transcription complex, subunit 10 (CNOT10), mRNA.	22:7186642-7244947	231
ENSBTAG00000015254	GLB1	Bos taurus galactosidase, beta 1 (GLB1), mRNA.	22:7413788-7517634	13
ENSBTAG00000004081	FAT3	FAT atypical cadherin 3	23:39906665-39927833	103
ENSBTAG00000020559	STMND1	stathmin domain containing 1	23:39906665-39927833	101
ENSBTAG00000001260	PINLYP	phospholipase A2 inhibitor and LY6/PLAUR domain containing	25:28339035-28445471	113
ENSBTAG00000000390	TPST1	Bos taurus tyrosylprotein sulfotransferase 1 (TPST1), mRNA.	25:28339035-28445471	123

ENSBTAG00000006212	GTF2IRD1	GTF2I repeat domain containing 1	25:33414265-33493476	27
ENSBTAG00000014698	CARD11	Bos taurus caspase recruitment domain family, member 11 (CARD11), mRNA.	25:40960556-41068746	25
ENSBTAG00000017871	GNA12	guanine nucleotide binding protein (G protein) alpha 12	25:41099259-41171209	64
ENSBTAG00000026194	AMZ1	archaelysin family metallopeptidase 1	25:41183195-41190361	26
ENSBTAG00000014502	BRAT1	BRCA1-associated ATM activator 1	25:41207310-41216105	28
ENSBTAG00000019297	GFRA1	Bos taurus GDNF family receptor alpha 1 (GFRA1), mRNA.	26:36789225-37020529	241
ENSBTAG00000018404	PRKG1	Bos taurus protein kinase, cGMP-dependent, type I (PRKG1), mRNA.	26:6906081-8343629	678
ENSBTAG00000021592	WRN	Bos taurus Werner syndrome, RecQ helicase-like (WRN), mRNA.	27:26263003-26449795	9
ENSBTAG00000045717	ADO	2-aminoethanethiol (cysteamine) dioxygenase	28:19004887-19005699	46
ENSBTAG00000046409	EGR2	early growth response 2	28:19012870-19015927	49
ENSBTAG00000017509	MYPN	myopalladin	28:24745815-24833215	160
ENSBTAG00000017521	ATOH7	atonal homolog 7 (Drosophila)	28:24845410-24845868	111
ENSBTAG00000014315	PBLD	Bos taurus phenazine biosynthesis-like protein domain containing (PBLD), mRNA.	28:24869418-24892252	67
ENSBTAG00000012667	CAMK2G	Bos taurus calcium/calmodulin-dependent protein kinase II gamma, mRNA.	28:29878540-29930559	29
ENSBTAG00000005947	PLAU	Bos taurus plasminogen activator, urokinase (PLAU), mRNA.	28:29964983-29971029	19
ENSBTAG00000010032	NTM	Bos taurus neurotrimin (NTM), mRNA.	29:35154689-35575203	79
ENSBTAG00000003202	OPRD1	opioid receptor, delta 1	29:36619711-36645779	88
ENSBTAG00000017602	TMEM45B	Bos taurus transmembrane protein 45B (TMEM45B), mRNA.	29:36619711-36645779	54
ENSBTAG00000017610	NFRKB	nuclear factor related to kappaB binding protein	29:36650181-36680357	47
ENSBTAG00000013942	DAGLA	diacylglycerol lipase, alpha	29:40857731-40883995	3
ENSBTAG00000009393	GAL	Bos taurus galanin prepropeptide (GAL), mRNA.	29:46759818-46765617	73
ENSBTAG00000021999	CPT1A	Uncharacterized protein	29:46822027-46862300	70
ENSBTAG00000022188	PPFIA1	protein tyrosine phosphatase, receptor type, f polypeptide (PTPRF), interacting protein (liprin), alpha 1	29:48078850-48152891	178
ENSBTAG00000006071	CTTN	Bos taurus cortactin (CTTN), mRNA.	29:48167168-48194210	83
ENSBTAG00000045502	Metazoa_SRP	Metazoan signal recognition particle RNA	X:17862231-17862527	124
ENSBTAG000000xxxxx	5S_rRNA	5S ribosomal RNA	multiple loci	2510
ENSBTAG0000004xx	7SK	7SK RNA	multiple loci	780
ENSBTAG0000004xxxx	SNORA31	Small nucleolar RNA SNORA31	multiple loci	218
ENSBTAG0000004xxxx	U5	U5 spliceosomal RNA	multiple loci	562
ENSBTAG0000004xxxx	U6	U6 spliceosomal RNA	multiple loci	6879
ENSBTAG0000004xxxx	U8	U8 spliceosomal RNA	multiple loci	187
ENSBTAG0000004xxxx	U11	U11 spliceosomal RNA	multiple loci	172

Table S7. Significant GO terms in biological process from the extracted genes of the correlation network constructed based on the MI and the XP-CLR (*P*-value less than 0.05).

GO:ID	GO terms	Associated genes	*P*-value*	FDR
GO:0046883	regulation of hormone secretion	HNF1A, FGF23, CPT1A	1.8E-03	2.4E-02
GO:0030278	regulation of ossification	CALCR, FGF23, CDK6	2.5E-03	3.4E-02
GO:0048878	chemical homeostasis	CALCR, HNF1A, EGR2, FGF23	1.2E-02	1.5E-01
GO:0030279	negative regulation of ossification	CALCR, FGF23	1.3E-02	1.7E-01
GO:0051046	regulation of secretion	HNF1A, FGF23, CPT1A	1.6E-02	2.0E-01
GO:0044093	positive regulation of molecular function	CALCR, EPB41, FGF23, HOMER1	1.7E-02	2.1E-01
GO:0060341	regulation of cellular localization	HNF1A, FGF23, CPT1A	2.3E-02	2.7E-01
GO:0042592	homeostatic process	CALCR, HNF1A, EGR2, FGF23	3.2E-02	3.6E-01
GO:0050796	regulation of insulin secretion	HNF1A, CPT1A	3.8E-02	4.1E-01
GO:0002791	regulation of peptide secretion	HNF1A, CPT1A	4.3E-02	4.6E-01
GO:0006873	cellular ion homeostasis	CALCR, EGR2, FGF23	4.9E-02	5.0E-01

* *P*-value is the modified Fisher exact *p*-value

Table S8. 117 identified genes based on MI and XP-EHH and the number of the identified SNPs.

Ensemble Gene ID	Gene Symbol	Description	Location	#SNP
ENSBTAG00000009115	SPSB4	splA/ryanodine receptor domain and SOCS box containing 4	1:128830908-128901497	8
ENSBTAG00000009942	PLCL2	Bos taurus phospholipase C-like 2 (PLCL2), mRNA.	1:155618262-155833445	32
ENSBTAG00000013806	EFHB	Bos taurus EF-hand domain family, member B (EFHB), mRNA.	1:44647591-44712979	4
ENSBTAG00000033690	BARD1	BRCA1 associated RING domain 1	2:103278272-103371020	5
ENSBTAG00000021050	ECEL1	endothelin converting enzyme-like 1	2:120925717-120933239	147
ENSBTAG00000013309	SFRS4	serine/arginine-rich splicing factor 4	2:124956594-124982238	26
ENSBTAG00000010498	SLC40A1	Bos taurus solute carrier family 40 (iron-regulated transporter), member 1, mRNA.	2:6716591-6740329	5
ENSBTAG00000005784	CSMD2	CUB and Sushi multiple domains 2	3:112558420-112898742	141
ENSBTAG00000037819	SFT2D2	Bos taurus SFT2 domain containing 2 (SFT2D2), mRNA.	3:329276-345806	6
ENSBTAG00000019033	CD84	CD84 molecule	3:9215138-9239453	15
ENSBTAG00000018185	ASB4	Bos taurus ankyrin repeat and SOCS box containing 4 (ASB4), mRNA.	4:12597838-12699701	115
ENSBTAG00000008129	CLSTN3	Bos taurus calsyntenin 3 (CLSTN3), mRNA.	5:103581388-103611327	30
ENSBTAG00000008127	RBP5	Bos taurus retinol binding protein 5, cellular (RBP5), mRNA.	5:103612392-103617657	31
ENSBTAG00000016204	C1RL	complement component 1, r subcomponent-like	5:103633960-103644323	29
ENSBTAG00000017378	GALNT8	polypeptide N-acetylgalactosaminyltransferase 8	5:105799704-105837839	197
ENSBTAG00000047347	RAD51AP1	RAD51 associated protein 1; Uncharacterized protein	5:105996438-106014092	5
ENSBTAG00000001057	ARFGAP3	Bos taurus ADP-ribosylation factor GTPase activating protein 3, mRNA.	5:114291727-114341661	37
ENSBTAG00000003021	SP1	Bos taurus Sp1 transcription factor (SP1), mRNA.	5:26769555-26804655	70
ENSBTAG00000033292	SP7	Bos taurus Sp7 transcription factor (SP7), mRNA.	5:26848177-26855849	28
ENSBTAG00000043962	SLC2A13	Bos taurus solute carrier family 2 (facilitated glucose transporter), member 13	5:41062652-41559936	31
ENSBTAG00000010644	CHST11	carbohydrate (chondroitin 4) sulfotransferase 11	5:68385645-68645774	92
ENSBTAG00000034693	SYT1	Bos taurus synaptotagmin I (SYT1), mRNA.	5:8836101-9062297	46
ENSBTAG00000008989	LOH12CR1	Bos taurus loss of heterozygosity, 12, chromosomal region 1 (LOH12CR1), mRNA.	5:97814581-97911805	21
ENSBTAG00000008721	MANSC1	Bos taurus MANSC domain containing 1 (MANSC1), mRNA.	5:97925815-97949862	22
ENSBTAG00000012343	TSPAN5	Bos taurus tetraspanin 5 (TSPAN5), mRNA.	6:27333479-27512867	160
ENSBTAG00000047743	KCNIP4	Bos taurus Kv channel interacting protein 4 (KCNIP4), mRNA.	6:41709362-43021626	381
ENSBTAG00000000061	PCDH7	protocadherin 7	6:51536863-52007783	267
ENSBTAG00000011156	ATP8A1	probable phospholipid-transporting ATPase IA	6:62893173-63128455	101
ENSBTAG00000025443	FNIP1	folliculin interacting protein 1	7:23993128-24063506	23
ENSBTAG00000003349	JAKMIP2	janus kinase and microtubule interacting protein 2	7:60809074-60998968	133
ENSBTAG00000043972	SLC24A2	solute carrier family 24 (sodium/potassium/calcium exchanger), member 2	8:24497953-24778056	19
ENSBTAG00000008204	C9orf41	chromosome 9 open reading frame 41	8:51344227-51383813	36
ENSBTAG00000003137	LPPR1	Bos taurus lipid phosphate phosphatase-related protein type 1 (LPPR1), mRNA.	8:92346242-92674692	209
ENSBTAG00000001826	SASH1	SAM and SH3 domain containing 1	9:86852607-86998307	40

ENSBTAG00000025853	HOMER1	Bos taurus homer homolog 1 (Drosophila) (HOMER1), mRNA.	10:10350692-10484916	100
ENSBTAG00000000218	MAP2K5	Bos taurus mitogen-activated protein kinase kinase 5 (MAP2K5), mRNA.	10:14354690-14626128	62
ENSBTAG00000009030	NOX5	Bos taurus NADPH oxidase, EF-hand calcium binding domain 5 (NOX5), mRNA.	10:15835507-15867515	131
ENSBTAG00000003915	LARP6	la-related protein 6	10:17662965-17682955	31
ENSBTAG00000000432	TRAC	T cell receptor alpha constant	10:22106909-23304334	73
ENSBTAG00000002781	GCNT4	glucosaminyl (N-acetyl) transferase 4, core 2	10:6432188-6433552	42
ENSBTAG00000011379	KCNH5	Bos taurus potassium voltage-gated channel, subfamily H (eag-related), member 5	10:75235434-75637242	47
ENSBTAG00000027080	SLC8A3	solute carrier family 8 (sodium/calcium exchanger), member 3	10:82128379-82273227	54
ENSBTAG00000019454	ANGEL1	angel homolog 1 (Drosophila)	10:89053688-89074011	5
ENSBTAG00000013181	LRRC74A	leucine rich repeat containing 74A, with synonyms of C10H14orf166B	10:89088878-89117693	10
ENSBTAG00000025324	NRXN3	neurexin 3	10:91597994-92223876	67
ENSBTAG00000004510	SARDH	sarcosine dehydrogenase	11:104578933-104634907	10
ENSBTAG00000032521	PLEKHH2	pleckstrin homology domain containing, family H (with MyTH4 domain) member 2	11:25974409-26070971	64
ENSBTAG00000032519	CAMKMT	calmodulin-lysine N-methyltransferase	11:26602922-27032485	82
ENSBTAG00000020614	PRKCE	protein kinase C, epsilon	11:27935104-28472632	117
ENSBTAG00000017448	EFEMP1	Bos taurus EGF containing fibulin-like extracellular matrix protein 1, mRNA.	11:38338744-38408288	94
ENSBTAG00000030046	bta-mir-216b	microRNA mir-216b	11:38511117-38511202	134
ENSBTAG00000005273	IL1R1	interleukin 1 receptor, type I	11:6876948-6929909	5
ENSBTAG00000031014	LDAH	Lipid droplet associated hydrolase, Chromosome 11 open reading frame (C2orf43)	11:78168945-78254093	134
ENSBTAG00000012827	TTLL11	TTLL11 protein; Uncharacterized protein	11:92737269-92903570	1
ENSBTAG00000013879	DGKH	diacylglycerol kinase, eta	12:12293695-12405237	66
ENSBTAG00000034222	CAB39L	calcium binding protein 39-like	12:19016368-19079171	182
ENSBTAG00000004595	GML	Bos taurus glycosylphosphatidylinositol anchored molecule like protein, mRNA.	14:2715416-2742638	64
ENSBTAG00000002192	C14H8orf46	Bos taurus chromosome 14 open reading frame, human C8orf46 (C14H8orf46)	14:32760708-32786980	6
ENSBTAG00000032657	TEAD1	TEA domain family member 1 (SV40 transcriptional enhancer factor)	15:40303805-40482346	47
ENSBTAG00000043981	SBF2	Bos taurus SET binding factor 2 (SBF2), mRNA.	15:42970987-43480821	12
ENSBTAG00000020989	SUSD4	sushi domain containing 4	16:27388710-27522155	35
ENSBTAG00000014191	QSOX1	quiescin Q6 sulfhydryl oxidase 1	16:62804447-62845817	4
ENSBTAG00000002531	ARHGAP10	Bos taurus Rho GTPase activating protein 10 (ARHGAP10), mRNA.	17:10182664-10560361	73
ENSBTAG00000016739	RASAL1	RAS protein activator like 1 (GAP1 like)	17:63397791-63429841	1
ENSBTAG00000011924	CCDC42B	coiled-coil domain containing 42B	17:63439743-63448400	2
ENSBTAG00000011930	DDX54	DEAD (Asp-Glu-Ala-Asp) box polypeptide 54	17:63448841-63466323	16
ENSBTAG00000003297	OASL	Bos taurus 2'-5'-oligoadenylate synthetase-like (OASL), mRNA.	17:65446987-65463409	13
ENSBTAG00000005367	VRK3	Bos taurus vaccinia related kinase 3 (VRK3), mRNA.	18:56748766-56784317	45
ENSBTAG00000012064	ZNF473	zinc finger protein 473	18:56790637-56796623	35
ENSBTAG00000013740	SP2	Bos taurus Sp2 transcription factor (SP2), mRNA.	19:39194570-39296546	68

ENSBTAG00000034681	CA10	Bos taurus carbonic anhydrase X (CA10), mRNA.	19:627558-1472951	47
ENSBTAG00000021292	ANKFN1	ankyrin-repeat and fibronectin type III domain containing 1	19:7329920-7490693	12
ENSBTAG00000017026	DEPDC1B	DEP domain containing 1B	20:18563739-18654726	1
ENSBTAG00000010510	DHX29	Bos taurus DEAH (Asp-Glu-Ala-His) box polypeptide 29 (DHX29), mRNA.	20:23853539-23900484	54
ENSBTAG00000047594	CCNO	cyclin O	20:23924721-23926909	2
ENSBTAG00000024801	RANBP17	RAN binding protein 17	20:2680574-3054892	3
ENSBTAG00000036426	bta-mir-377	mircroRNA mir-377, member of mir-154 family	21: 67600490-67600558	2
ENSBTAG00000036426	bta-mir-541	mircroRNA mir-541, member of mir-154 family	21: 67602344-67602427	2
ENSBTAG00000036426	bta-mir-3957	mircroRNA mir-3957, member of mir-154 family	21: 67602730-67602806	2
ENSBTAG00000036426	bta-mir-409b	mircroRNA mir-409b, member of mir-154 family	21: 67603246-67603324	2
ENSBTAG00000036426	bta-mir-412	mircroRNA mir-412, member of mir-154 family	21: 67603388-67603477	2
ENSBTAG00000036426	bta-mir-369	mircroRNA mir-369, member of mir-154 family	21: 67603541-67603609	2
ENSBTAG00000036426	bta-mir-410	mircroRNA mir-410, member of mir-154 family	21: 67603865-67603945	2
ENSBTAG00000036426	bta-mir-656	mircroRNA mir-656, member of mir-154 family	21: 67604689-67604767	2
ENSBTAG00000017719	AKAP6	A kinase (PRKA) anchor protein 6	21:43132861-43630707	510
ENSBTAG00000004679	WARS	Bos taurus tryptophanyl-tRNA synthetase (WARS), mRNA.	21:66888332-66916535	32
ENSBTAG00000007233	WDR25	WD repeat domain 25	21:66917780-67066000	93
ENSBTAG00000016533	FOXP1	Bos taurus forkhead box P1 (FOXP1), mRNA.	22:30411677-30803681	310
ENSBTAG00000044062	RBMS3	RNA binding motif, single stranded interacting protein 3	22:3899285-4695113	39
ENSBTAG00000000024	DYM	dymeclin	24:49193606-49590504	647
ENSBTAG00000005738	ATP8B1	Bos taurus ATPase, aminophospholipid transporter, class I, type 8B, member 1	24:57380076-57487773	166
ENSBTAG00000005917	PARD6G	Bos taurus par-6 partitioning defective 6 homolog gamma (C. elegans) (PARD6G)	24:582652-659851	139
ENSBTAG00000006212	GTF2IRD1	GTF2I repeat domain containing 1	25:33414265-33493476	27
ENSBTAG00000011818	COL26A1	collagen, type XXVI, alpha 1	25:35776553-35960746	28
ENSBTAG00000014698	CARD11	Bos taurus caspase recruitment domain family, member 11 (CARD11), mRNA.	25:40960556-41068746	25
ENSBTAG00000017871	GNA12	guanine nucleotide binding protein (G protein) alpha 12	25:41099259-41171209	64
ENSBTAG00000007474	EIF3B	Bos taurus eukaryotic translation initiation factor 3, subunit B (EIF3B), mRNA.	25:41435744-41453953	7
ENSBTAG00000019297	GFRA1	Bos taurus GDNF family receptor alpha 1 (GFRA1), mRNA.	26:36789225-37020529	241
ENSBTAG00000018404	PRKG1	Bos taurus protein kinase, cGMP-dependent, type I (PRKG1), mRNA.	26:6906081-8343629	678
ENSBTAG00000021133	TENM3	teneurin transmembrane protein 3	27:12619929-12807702	2
ENSBTAG00000004613	CSGALNACT1	chondroitin sulfate N-acetylgalactosaminyltransferase 1	27:37841210-38016326	18
ENSBTAG00000006729	ARID5B	AT rich interactive domain 5B (MRF1-like)	28:18003736-18191994	65
ENSBTAG00000045717	ADO	2-aminoethanethiol (cysteamine) dioxygenase	28:19004887-19005699	46
ENSBTAG00000046409	EGR2	Uncharacterized protein	28:19012870-19015927	49
ENSBTAG00000012667	CAMK2G	Bos taurus calcium/calmodulin-dependent protein kinase II gamma, mRNA.	28:29878540-29930559	29
ENSBTAG00000010032	NTM	Bos taurus neurotrimin (NTM), mRNA.	29:35154689-35575203	79

ENSBTAG00000003967	OTUB1	Bos taurus OTU domain, ubiquitin aldehyde binding 1 (OTUB1), mRNA.	29:42882593-42889928	32
ENSBTAG00000017181	MACROD1	Bos taurus MACRO domain containing 1 (MACROD1), mRNA.	29:42890800-43092842	251
ENSBTAG000000xxxxx	5S_rRNA	5S ribosomal RNA	multiple loci	2510
ENSBTAG0000004xxxx	SNORA31	Small nucleolar RNA SNORA31	multiple loci	218
ENSBTAG0000004xxxx	SNORA5	Small nucleolar RNA SNORA5	multiple loci	127
ENSBTAG0000004xxxx	SNORD112	Small nucleolar RNA SNORD112	multiple loci	374
ENSBTAG0000004xxxx	SNORD113	Small nucleolar RNA SNORD113/SNORD114 family	multiple loci	421
ENSBTAG000000xxxxx	U1	U1 spliceosomal RNA	multiple loci	138
ENSBTAG000000xxxxx	U2	U2 spliceosomal RNA	multiple loci	490
ENSBTAG0000004xxxx	U4	U4 spliceosomal RNA	multiple loci	78
ENSBTAG0000004xxxx	U6	U6 spliceosomal RNA	multiple loci	6878

Table S9. Significant GO terms in biological process from the extracted genes of the correlation network constructed based on the MI and the XP-EHH (*P*-value less than 0.05).

GO:ID	GO terms	Associated genes	*P*-value*	FDR
GO:0002520	immune system development	CARD11, SP1, FOXP1	2.1E-03	2.5E-02
GO:0006357	regulation of transcription from RNA polymerase II promoter	SP1, SP7, FOXP1	7.4E-03	8.6E-02
GO:0060348	bone development	SP1, SP7	3.0E-02	3.1E-01
GO:0001503	ossification	SP1, SP7	3.0E-02	3.1E-01

* *P*-value is the modified Fisher exact *p*-value

Table S10. Number of significant missense and nonsense mutations of the identified genes in N'Dama breed. UTR, untranslated region; N.C., not clear.

Annotated genomic locations	Coding effects	wMI	MI ∩ XP-CLR	MI ∩ XP-EHH
Exon	Missense	1	12	7
Exon	Nonsense	0	0	1
Exon	Synonymous	5	24	16
Downstream	N.C.	11	106	132
Intron	N.C.	2617	2897	4032
Splice site region	N.C.	0	1	0
3'-UTR	N.C.	0	1	3
5'-UTR	N.C.	0	1	0

Table S11. Description of the identified genes including N'Dama-specific missense and nonsense mutations and the related function.

Proposed model	Gene	Ensemble Gene ID	Description	Location	Related function [ref.]
**wMI**	USH2A	ENSBTAG00000006188	Usher syndrome 2A (autosomal recessive, mild)	16:19573856-20502175	nervous system [1]
**MI ∩ XP-CLR**	ACAD9	ENSBTAG00000003242	acyl-CoA dehydrogenase family member 9, mitochondrial	22:59573116-59609270	cellular metabolic system [2, 3]
	AMZ1	ENSBTAG00000026194	archaelysin family metallopeptidase 1	25:41183195-41190361	nervous system [4]
	CDADC1	ENSBTAG00000002271	cytidine and dCMP deaminase domain containing 1	12:18962979-18990944	cellular metabolic system [5]
	EML1	ENSBTAG00000013491	echinoderm microtubule associated protein like 1	21:66358950-66556801	nervous system [6, 7]
	EOMES	ENSBTAG00000016920	eomesodermin	22:2112410-2117440	immune/nervous system [8, 9, 10]
	OPCML	ENSBTAG00000020769	opioid binding protein/cell adhesion molecule-like	29:34554780-35085038	nervous system [11]
	PIK3C2G	ENSBTAG00000020715	phosphatidylinositol-4-phosphate 3-kinase, catalytic subunit type 2 gamma	5:91835146-92276939	cellular metabolic system [12]
	SLIT3	ENSBTAG00000017746	slit guidance ligand 3	20:324518-507045	nervous system [13, 14]
	TIGAR	ENSBTAG00000016650	TP53 induced glycolysis regulatory phosphatase	5:106223071-106238040	cellular metabolic system [15]
	TPST1	ENSBTAG00000000390	tyrosylprotein sulfotransferase 1	25:28339035-28445471	immune system [16]
**MI ∩ XP-EHH**	C1RL	ENSBTAG00000016204	complement component 1, r subcomponent-like	5:103633960-103644323	immune system [17]
	DDX54	ENSBTAG00000011930	DEAD (Asp-Glu-Ala-Asp) box polypeptide 54	17:63448841-63466323	nervous system [18]
	NOX5	ENSBTAG00000009030	NADPH oxidase, EF-hand calcium binding domain 5	10:15835507-15867515	cellular metabolic system [19]
	RANBP17	ENSBTAG00000024801	RAN binding protein 17	20:2680574-3054892	Immune system [20, 21]
	SBF2	ENSBTAG00000043981	SET binding factor 2	15:42970987-43480821	nervous system [22,23,24]

Table S12. Identified genes based on XP-CLR tests between N'Dama and Ogaden breeds (*p*-value < 1.0e-2). Chr, chromosome.

Chr	Window (Mbp)	SNPs	XP-CLR	Genes in XP-CLR regions
**1**	58775238-58825238	371	245.5078	KIAA2018
**1**	155625238-155675238	600	257.2767	PLCL2
**1**	136975238-137025238	600	329.4092	U8
**1**	137075238-137125238	600	229.5541	BFSP2,TMEM108
**2**	124975208-125025208	318	224.6311	EPB41,SFRS4,TMEM200B
**2**	122725208-122775208	600	256.439	FABP3
**2**	125225208-125275208	507	255.226	OPRD1
**2**	6725208-6775208	600	353.9891	SLC40A1
**2**	104525208-104575208	600	270.0336	MREG
**2**	126725208-126775208	600	226.3262	SLC9A1
**2**	6175208-6225208	600	273.6079	MSTN
**2**	6875208-6925208	597	230.2628	WDR75
**2**	30275208-30325208	594	290.8528	SCN1A
**2**	125025208-125075208	483	289.7518	EPB41
**2**	127775208-127825208	574	255.9709	AUNIP,PAQR7,STMN1
**2**	129025208-129075208	600	250.3273	NIPAL3
**2**	122625208-122675208	530	233.4238	HCRTR1,PEF1
**3**	113925115-113975115	396	226.7295	UGT1A6
**3**	106875115-106925115	600	244.4363	PPIE
**3**	3025115-3075115	600	249.643	UCK2
**3**	114125115-114175115	600	267.9319	HJURP
**3**	112925115-112975115	600	351.9611	ZSCAN20
**3**	10625115-10675115	543	325.7138	CADM3,DARC
**3**	108725115-108775115	490	283.9935	EPHA10
**3**	89725115-89775115	507	233.8999	C8B
**3**	116375115-116425115	600	270.7609	IQCA1
**3**	114075115-114125115	529	317.4321	HJURP,MROH2A
**4**	10575207-10625207	600	234.6748	CALCR
**4**	1225207-1275207	600	263.4065	5S_rRNA
**4**	19075207-19125207	549	229.2991	PHF14
**4**	18975207-19025207	600	238.0823	NDUFA4,PHF14
**4**	10025207-10075207	357	307.4635	CDK6
**4**	114725207-114775207	600	245.5519	CRYGN,WDR86
**4**	114625207-114675207	587	254.5437	SMARCD3
**5**	114475606-114525606	584	378.5148	TTLL1
**5**	92225606-92275606	600	224.8831	PIK3C2G
**5**	101775606-101825606	490	227.0173	DPPA3
**5**	105775606-105825606	600	276.826	GALNT8
**5**	11075606-11125606	600	345.0202	PPFIA2
**5**	113925606-113975606	600	224.2576	RRP7A,SERHL2
**5**	106175606-106225606	544	230.8478	FGF23,TIGAR
**5**	1125606-1175606	600	229.7213	LGR5,U6
**5**	78575606-78625606	493	228.5408	AMN1
**5**	113575606-113625606	404	322.3037	MGC127055,MGC152344,NDUFA6
**5**	112525606-112575606	476	241.4068	SLC25A17
**5**	114275606-114325606	600	307.0753	ARFGAP3
**5**	44675606-44725606	600	295.0912	LYZ,U6
**5**	99025606-99075606	600	276.7876	TAS2R42,TAS2R46
**5**	30675606-30725606	348	233.661	PRPH
**5**	112175606-112225606	323	331.4064	ADSL
**5**	94875606-94925606	555	225.8883	PTPRO

**5**	31325606-31375606	600	239.0465	LALBA
**6**	27325474-27375474	547	243.9898	TSPAN5,U6
**6**	87425474-87475474	600	312.2601	CABS1
**6**	27425474-27475474	598	393.452	TSPAN5
**6**	43525474-43575474	600	322.8688	GPR125
**6**	27375474-27425474	600	306.8198	TSPAN5
**6**	16675474-16725474	593	262.6255	LRIT3,RPS26
**7**	24325070-24375070	600	275.0564	CDC42SE2
**7**	23875070-23925070	379	242.9359	ACSL6
**8**	112677553-112727553	512	313.6158	STOM
**8**	87077553-87127553	600	248.477	CDC42BPA
**8**	51377553-51427553	430	234.967	C9orf41,NMRK1
**8**	112827553-112877553	600	331.7333	ALLC,COLEC11,DCDC2C
**8**	112627553-112677553	574	372.0268	GSN,STOM
**8**	92677553-92727553	590	290.0551	BAAT
**8**	106577553-106627553	407	278.6989	U6
**9**	98325194-98375194	600	259.0355	AGPAT4
**9**	23825194-23875194	600	266.3963	SNAP91
**9**	84225194-84275194	464	242.1069	SHPRH
**9**	9575194-9625194	471	226.4154	COL9A1
**9**	96725194-96775194	600	235.9407	RSPH3
**9**	96775194-96825194	600	270.6649	RSPH3,TAGAP
**10**	10426785-10476785	600	311.686	HOMER1
**10**	82226785-82276785	600	286.4388	SLC8A3
**10**	9326785-9376785	600	236.7985	SCAMP1
**10**	44326785-44376785	574	311.9893	FRMD6
**10**	10476785-10526785	489	230.9345	HOMER1
**10**	14376785-14426785	441	237.6634	MAP2K5
**10**	12226785-12276785	415	245.4558	IGDCC3,IGDCC4
**10**	28626785-28676785	600	287.875	AVEN
**10**	12176785-12226785	500	266.459	IGDCC3,U5,U5
**10**	28726785-28776785	600	238.4167	AVEN
**10**	44826785-44876785	548	275.3926	GNG2
**10**	35626785-35676785	552	287.9598	GPR176
**11**	28079718-28129718	600	275.8979	PRKCE
**11**	6979718-7029718	571	264.4801	5S_rRNA,IL1RL2
**11**	6929718-6979718	566	231.8386	IL1R1
**11**	25829718-25879718	552	267.026	THADA
**11**	7079718-7129718	600	285.4231	IL1RL1
**11**	26029718-26079718	600	230.3765	DYNC2LI1,PLEKHH2
**11**	63529718-63579718	600	239.0432	ACTR2
**11**	25979718-26029718	453	232.8285	PLEKHH2
**11**	329718-379718	600	315.6985	MERTK
**11**	44229718-44279718	600	229.8865	SH3RF3
**11**	48829718-48879718	495	383.5963	ST3GAL5
**11**	9629718-9679718	544	244.7664	NK1R
**12**	33577182-33627182	600	270.6043	5S_rRNA,SHISA2
**12**	19227182-19277182	557	300.9702	ARL11,RCBTB1
**12**	18977182-19027182	481	241.0201	CAB39L,CDADC1
**12**	53277182-53327182	425	232.7512	EDNRB
**13**	36925828-36975828	387	236.6827	ARMC4
**13**	76175828-76225828	576	278.6647	U6
**14**	77025240-77075240	600	362.8919	MMP-16
**14**	2825240-2875240	522	264.3637	LYPD2,PSCA,SLURP1,THEM6
**14**	2725240-2775240	548	362.1478	GML,LY6K

**15**	59125747-59175747	493	263.8749	BDNF
**15**	36825747-36875747	518	242.9682	SOX6
**15**	66425747-66475747	496	250.7867	CD44
**15**	39175747-39225747	513	265.1479	SPON1
**16**	67375129-67425129	600	245.2286	FAM129A
**16**	57775129-57825129	600	252.2302	TNN
**16**	27375129-27425129	600	232.2523	SUSD4
**17**	73427000-73477000	508	251.8027	GGT5
**17**	65427000-65477000	466	281.9122	C12orf43,HNF1A,OASL
**17**	63477000-63527000	592	239.4873	IQCD
**17**	29177000-29227000	600	311.7802	SCLT1
**18**	47228128-47278128	600	257.1945	ZNF566
**18**	52078128-52128128	600	400.5712	PINLYP,XRCC1
**18**	64178128-64228128	600	411.9138	MGC157368
**18**	53878128-53928128	309	233.2531	NOVA2
**18**	63478128-63528128	600	405.9455	TARM1
**18**	63828128-63878128	600	246.5637	ZNF667
**18**	65778128-65828128	595	236.4943	A1BG,ZNF8
**18**	56728128-56778128	420	253.9884	ATF5,VRK3
**19**	3875142-3925142	534	379.5811	KIF2B,SNORA31
**19**	50875142-50925142	588	468.7558	bta-mir-2345
**19**	39725142-39775142	438	234.4341	ARHGAP23
**19**	24525142-24575142	600	389.9555	U6
**19**	12325142-12375142	464	230.8311	BCAS3
**19**	50925142-50975142	557	265.1	bta-mir-2345,bta-mir-2345
**19**	61225142-61275142	600	233.4464	KCNJ16
**20**	475024-525024	600	230.8586	SLIT3
**21**	30725020-30775020	582	295.2072	OTUD7A
**21**	66475020-66525020	600	326.3721	EMAPL
**21**	43625020-43675020	508	325.6471	AKAP6
**22**	59176138-59226138	600	252.879	NUP210
**22**	61126138-61176138	587	286.5962	CHST13,UROC1,ZXDC
**22**	7376138-7426138	558	248.3043	CCR4,GLB1,U11
**22**	59876138-59926138	527	291.036	RAB7A
**22**	23226138-23276138	600	231.5577	CRBN,TRNT1
**22**	2076138-2126138	538	300.6741	EOMES
**22**	61026138-61076138	565	237.1176	CHCHD6
**22**	60926138-60976138	501	310.893	CHCHD6,PLXNA1
**22**	42276138-42326138	600	285.8827	Metazoa_SRP
**22**	61226138-61276138	600	276.1726	ALDH1L1
**22**	7226138-7276138	554	279.2796	CNOT10
**22**	59526138-59576138	495	246.436	ACAD9,IQSEC1
**22**	4276138-4326138	600	301.0903	RBMS3
**22**	55076138-55126138	600	254.1008	ATP2B2
**23**	34525665-34575665	528	444.9955	PRP1,U6
**23**	27725665-27775665	596	296.4908	POU5F1
**23**	39875665-39925665	600	253.1559	STMND1
**24**	49725722-49775722	529	254.4852	bta-mir-2381
**24**	50675722-50725722	595	230.0774	MAPK4
**24**	51175722-51225722	592	275.0448	AP3S1
**24**	25725722-25775722	600	227.4236	B4GALT6
**25**	41075036-41125036	600	272.5385	GNA12
**25**	41375036-41425036	600	231.8153	CHST12
**25**	40975036-41025036	600	227.1393	CARD11
**25**	13125036-13175036	425	224.9181	MKL2

**25**	28425036-28475036	509	296.4824	KCTD7,TPST1
**25**	33375036-33425036	412	253.8684	GTF2IRD1
**25**	41175036-41225036	560	347.4666	AMZ1,BRAT1
**26**	36825563-36875563	600	284.6551	GFRA1
**26**	7125563-7175563	600	438.5719	PRKG1
**26**	36875563-36925563	561	318.6714	GFRA1
**26**	7075563-7125563	600	311.3741	PRKG1
**26**	49525563-49575563	478	256.6242	EBF3
**27**	19927164-19977164	600	335.4889	MSR1
**27**	26277164-26327164	600	274.9313	WRN
**27**	327164-377164	600	296.1329	MYOM2
**28**	29875591-29925591	349	247.2128	CAMK2G
**28**	18375591-18425591	600	312.4676	RTKN2
**28**	19725591-19775591	516	232.6472	7SK,REEP3
**28**	29925591-29975591	418	248.7287	CAMK2G,PLAU
**28**	18975591-19025591	486	284.6605	ADO,EGR2
**28**	24825591-24875591	591	273.5349	ATOH7,MYPN,PBLD
**29**	35175001-35225001	587	227.3853	NTM
**29**	46725001-46775001	526	263.2557	GAL
**29**	35225001-35275001	600	374.3719	NTM
**29**	2525001-2575001	600	330.0098	FAT3,U6
**29**	46825001-46875001	600	306.7489	CPT1A
**29**	34525001-34575001	600	229.7251	OPCML
**29**	48125001-48175001	600	229.6791	CTTN,PPFIA1
**29**	36625001-36675001	387	228.1121	NFRKB,TMEM45B
**29**	34825001-34875001	583	230.9403	OPCML
**29**	40825001-40875001	557	259.8988	DAGLA
**29**	2425001-2475001	600	241.6156	FAT3

Table S13. Identified genes based on XP-EHH tests between N'Dama and Ogaden breeds (*p*-value < 1.0e-2). The value of XP-EHH is the maximum (positive) XP-EHH score of all SNPs within a window.

Chr	Window (Mbp)	SNPs	XP-EHH	Genes in XP-EHH regions
**1**	155600000-155650000	728	3.45028	PLCL2
**1**	128800000-128850000	849	3.75452	SPSB4
**1**	44650000-44700000	744	4.01343	EFHB
**2**	120900000-120950000	724	4.40738	ECEL1
**2**	6700000-6750000	807	3.52813	SLC40A1
**2**	122200000-122250000	330	4.00034	KHDRBS1,TMEM39B
**2**	127750000-127800000	737	4.16285	STMN1
**2**	98300000-98350000	513	3.68284	KANSL1L
**2**	103250000-103300000	906	3.47784	BARD1
**2**	121200000-121250000	514	4.24181	A3GALT2,PHC2,ZNF362
**2**	124950000-125000000	426	3.53507	SFRS4
**2**	122250000-122300000	475	3.64738	KHDRBS1
**3**	51550000-51600000	477	3.37583	BRDT,BRDT
**3**	112750000-112800000	774	3.82029	CSMD2
**3**	15550000-15600000	425	3.43855	ADAM15,EFNA3,EFNA4
**3**	113150000-113200000	446	3.29179	GIGYF2
**3**	113100000-113150000	387	3.29317	GIGYF2,KCNJ13
**3**	300000-350000	818	3.43411	SFT2D2
**3**	9200000-9250000	426	2.97913	CD84
**4**	40750000-40800000	844	3.78995	GNAT3
**4**	12550000-12600000	843	3.68393	ASB4
**5**	105950000-106000000	769	3.46645	DYRK4,RAD51AP1
**5**	68900000-68950000	609	4.1022	ALDH1L2
**5**	26800000-26850000	326	2.94649	SP1,SP7
**5**	9050000-9100000	841	3.8142	SYT1
**5**	28000000-28050000	430	3.10197	ACVR1B,GRASP
**5**	103600000-103650000	631	3.60436	C1RL,CLSTN3,RBP5
**5**	41550000-41600000	545	3.64313	SLC2A13
**5**	114250000-114300000	755	3.51368	ARFGAP3
**5**	105900000-105950000	452	2.92732	AKAP3,DYRK4
**5**	97900000-97950000	623	3.97603	LOH12CR1,MANSC1
**5**	30650000-30700000	518	3.53003	C1QL4,PRPH,TROAP
**5**	105800000-105850000	807	3.53045	GALNT8
**5**	68400000-68450000	488	3.41814	CHST11
**5**	26850000-26900000	383	2.99701	AAAS,C5H12orf10,ESPL1,PFDN5,SP7
**5**	68450000-68500000	675	3.66248	CHST11
**5**	9150000-9200000	628	3.63811	PAWR,SNORA5
**5**	113450000-113500000	663	3.53761	CENPM,SEPT3
**5**	118100000-118150000	1381	3.92662	TBC1D22A
**6**	27400000-27450000	877	4.10416	TSPAN5
**6**	41950000-42000000	795	3.46409	KCNIP4
**6**	26200000-26250000	489	3.19119	DAPP1
**6**	86200000-86250000	768	4.23269	5S_rRNA
**6**	85850000-85900000	396	2.92923	YTHDC1
**6**	51500000-51550000	477	3.25027	PCDH7
**6**	63100000-63150000	708	3.43112	ATP8A1
**6**	50200000-50250000	900	3.54915	5S_rRNA
**6**	68850000-68900000	433	3.11539	FRYL
**7**	17200000-17250000	728	3.90286	U4,ZNF558
**7**	60850000-60900000	782	4.10043	JAKMIP2
**7**	100400000-100450000	546	3.46621	RGMB

**7**	24000000-24050000	459	2.94065	FNIP1,U6
**7**	72350000-72400000	496	3.15145	EBF1
**7**	4450000-4500000	684	4.16087	KLHL26,U6
**8**	112650000-112700000	691	3.65977	STOM
**8**	105500000-105550000	495	3.36941	ATP6V1G1,C9orf91
**8**	48500000-48550000	419	3.27063	C8H9ORF85,SNORA65
**8**	92650000-92700000	811	3.62472	BAAT,LPPR1
**8**	106600000-106650000	713	3.82897	U6
**8**	24700000-24750000	734	3.46979	SLC24A2
**8**	105450000-105500000	436	3.20051	ATP6V1G1
**8**	6600000-6650000	631	3.61627	HPGD,U1
**8**	92700000-92750000	793	4.11166	MRPL50
**8**	48450000-48500000	440	3.00928	C8H9ORF85,FAM108B1
**8**	51350000-51400000	628	3.77929	C9orf41
**8**	51400000-51450000	635	3.8574	NMRK1,OSTF1
**8**	21750000-21800000	578	3.98875	DMRTA1
**9**	53350000-53400000	699	3.89521	KLHL32,MMS22L
**9**	34200000-34250000	840	4.59236	RFX6
**9**	84800000-84850000	471	3.01419	GRM1
**9**	86900000-86950000	762	3.47697	SASH1
**9**	96750000-96800000	740	4.03789	RSPH3
**10**	59600000-59650000	389	2.98234	AP4E1
**10**	89050000-89100000	656	3.87991	ANGEL1,C14orf166B
**10**	17650000-17700000	931	3.45971	LARP6
**10**	6400000-6450000	929	3.46306	GCNT4
**10**	91950000-92000000	970	3.87154	NRXN3
**10**	10450000-10500000	819	3.5446	HOMER1
**10**	80050000-80100000	460	3.40686	ARG2,U6
**10**	91900000-91950000	752	3.58271	NRXN3
**10**	14400000-14450000	795	3.70607	MAP2K5
**10**	35650000-35700000	942	3.93824	GPR176
**10**	82150000-82200000	843	4.13702	5S_rRNA,SLC8A3
**10**	36100000-36150000	406	3.07069	C10H15ORF23,DISP2,IVD
**10**	75600000-75650000	833	3.68678	KCNH5
**10**	44400000-44450000	681	3.47202	FRMD6
**10**	15850000-15900000	1062	3.9777	NOX5
**10**	22250000-22300000	890	3.52191	TRAC
**10**	35600000-35650000	774	3.50557	GPR176
**11**	27450000-27500000	690	3.81725	U6
**11**	26950000-27000000	396	2.92679	CAMKMT
**11**	38500000-38550000	493	3.31864	bta-mir-216b
**11**	78150000-78200000	538	3.46828	C2ORF43
**11**	92750000-92800000	924	4.05367	TTLL11
**11**	25900000-25950000	761	3.56915	THADA
**11**	92850000-92900000	756	3.56905	TTLL11
**11**	38300000-38350000	422	3.21187	EFEMP1
**11**	30800000-30850000	752	3.56289	GTF2A1L,LHCGR
**11**	92800000-92850000	957	4.01745	TTLL11
**11**	25800000-25850000	612	3.94678	THADA
**11**	6850000-6900000	729	3.46775	IL1R1
**11**	104550000-104600000	1105	3.88265	DBH,SARDH
**11**	28300000-28350000	499	3.28104	PRKCE
**11**	28350000-28400000	540	3.5736	PRKCE
**11**	26000000-26050000	727	4.08568	PLEKHH2
**11**	28450000-28500000	636	3.63063	PRKCE

**12**	12350000-12400000	780	3.74069	DGKH
**12**	19000000-19050000	778	3.51642	CAB39L
**13**	79350000-79400000	635	3.90472	FAM65C
**13**	60650000-60700000	634	3.47618	RSPO4
**14**	39150000-39200000	425	3.9024	U6
**14**	2700000-2750000	774	4.37582	GML
**14**	32750000-32800000	624	3.77562	C14H8orf46
**14**	37550000-37600000	483	2.99339	MSC
**14**	25550000-25600000	653	3.60125	IMPAD1
**14**	47150000-47200000	796	3.66242	MAL2
**14**	32800000-32850000	465	3.22391	MYBL1,U6
**15**	59150000-59200000	506	3.92468	BDNF
**15**	40450000-40500000	582	3.5323	TEAD1
**15**	52150000-52200000	670	3.47807	CHRNA10,NUP98
**15**	43250000-43300000	457	2.97492	SBF2
**16**	11300000-11350000	959	3.52071	U2
**16**	27350000-27400000	794	3.49532	SUSD4
**16**	62800000-62850000	620	3.79867	QSOX1
**16**	40400000-40450000	427	3.00136	DNM3
**17**	49450000-49500000	911	3.73789	TMEM132C
**17**	6100000-6150000	1022	4.07213	FAM160A1
**17**	65450000-65500000	584	3.54307	OASL
**17**	10200000-10250000	833	3.85742	ARHGAP10
**17**	63400000-63450000	640	3.57015	CCDC42B,DDX54,RASAL1
**18**	39800000-39850000	887	3.47284	CALB2
**18**	24150000-24200000	568	3.64285	MT4
**18**	56750000-56800000	612	3.45154	VRK3,ZNF473
**19**	3850000-3900000	901	3.86403	KIF2B,SNORA31
**19**	43150000-43200000	409	3.887	PTRF
**19**	800000-850000	793	4.0753	CA10
**19**	7300000-7350000	801	3.5732	ANKFN1
**19**	56600000-56650000	694	3.59152	LLGL2
**19**	39150000-39200000	477	3.10323	PNPO,PRR15L,SP2
**19**	24550000-24600000	1482	3.71419	U6
**20**	2650000-2700000	480	2.95447	RANBP17
**20**	23900000-23950000	737	3.43502	CCNO,DHX29,MCIDAS
**20**	18600000-18650000	654	3.46484	DEPDC1B
**21**	31050000-31100000	448	3.61283	ACSBG1,IDH3A
**21**	56150000-56200000	906	3.62911	SNORD113
**21**	46400000-46450000	483	3.19683	BRMS1L,U6
**21**	66900000-66950000	654	3.46513	WARS,WDR25
**21**	2800000-2850000	834	3.57177	ATP10A
**21**	43500000-43550000	560	3.50243	AKAP6
**21**	66700000-66750000	662	3.43949	DEGS2,EVL,bta-mir-342
**21**	43550000-43600000	889	3.75021	AKAP6
**21**	67600000-67650000	608	3.43211	bta-mir-369,bta-mir-377,bta-mir-3957,bta-mir-409b,bta-mir-410, bta-mir-412,bta-mir-541,bta-mir-656
**22**	1450000-1500000	997	3.62318	NEK10,SEC61G
**22**	57100000-57150000	470	3.00656	RAF1
**22**	30400000-30450000	482	3.06195	FOXP1
**22**	4650000-4700000	895	3.55475	RBMS3
**24**	22450000-22500000	764	4.0642	DTNA
**24**	50700000-50750000	882	3.53736	MAPK4
**24**	58200000-58250000	783	3.55535	ALPK2

**24**	25300000-25350000	820	3.5086	MEP1B
**24**	49450000-49500000	336	3.15667	DYM
**24**	58250000-58300000	611	3.61837	MALT1
**24**	22400000-22450000	689	4.18469	DTNA
**24**	650000-700000	418	3.53678	ADNP2,PARD6G
**24**	57350000-57400000	797	4.03102	ATP8B1,U6
**25**	41050000-41100000	975	3.63425	CARD11,GNA12
**25**	41100000-41150000	810	3.4995	GNA12
**25**	41400000-41450000	753	3.60358	EIF3B
**25**	40950000-41000000	815	3.47245	CARD11
**25**	28600000-28650000	870	3.49687	SBDS,TYW1
**25**	35800000-35850000	883	3.76034	COL26A1
**25**	700000-750000	601	3.71793	LMF1
**25**	750000-800000	548	3.70018	LMF1,SOX8
**25**	33450000-33500000	938	3.44265	GTF2IRD1
**26**	36800000-36850000	881	3.49299	GFRA1
**26**	7150000-7200000	775	3.88634	PRKG1
**26**	39200000-39250000	645	3.56232	PRLHR
**27**	19950000-20000000	691	3.573	MSR1
**27**	12650000-12700000	760	3.47139	TENM3
**27**	20000000-20050000	761	3.74069	MSR1
**27**	37800000-37850000	945	3.67134	CSGALNACT1
**28**	18100000-18150000	738	3.5169	ARID5B
**28**	18350000-18400000	753	3.78827	RTKN2
**28**	29850000-29900000	437	4.05983	CAMK2G,CHCHD1,NDST2,ZSWIM8
**28**	19000000-19050000	665	3.70093	ADO,EGR2
**29**	35200000-35250000	704	3.66634	NTM
**29**	17300000-17350000	869	3.52333	TENM4
**29**	35250000-35300000	923	3.6071	NTM
**29**	38650000-38700000	953	3.44129	PAG-15
**29**	49350000-49400000	564	3.67174	CDKN1C,SLC22A18
**29**	27150000-27200000	1095	4.18827	VWA5A
**29**	42850000-42900000	361	3.4319	MACROD1,MARK2,OTUB1,RCOR2, bta-mir-2406
**X**	13400000-13450000	939	4.1377	SMARCA1,SNORD112
**X**	50900000-50950000	732	3.66795	SRPX2,TSPAN6

Ratio of the extracted SNPs to all the SNPs on each chromosome

Chr.	1	2	3	4	5	6	7	8	9	10	11	12	13	14	15	16	17	18	19	20	21	22	23	24	25	26	27	28	29	X
I(A:N)	2.208	1.998	2.062	2.400	1.949	2.244	2.083	2.283	2.201	2.047	1.933	1.916	2.220	2.237	2.066	2.022	2.279	1.913	1.650	2.166	2.056	1.958	2.508	2.310	1.860	2.281	2.557	2.229	1.951	2.890
I(B:N)	2.473	2.110	2.134	2.148	1.866	2.431	2.140	2.404	2.452	2.159	2.054	2.193	2.449	2.395	2.448	2.351	2.354	1.921	1.917	2.535	2.164	1.860	2.758	2.122	1.865	2.078	2.624	2.414	2.076	1.882
I(K:N)	2.432	1.979	1.909	2.180	2.029	2.462	2.142	2.449	2.406	2.239	1.985	2.213	2.284	2.759	2.215	2.248	2.306	1.885	1.983	2.490	2.317	1.949	2.895	2.217	1.952	2.285	2.602	2.496	2.110	1.743
I(O:N)	2.361	1.945	2.041	2.221	1.784	2.311	2.190	2.299	2.327	2.001	1.975	2.097	2.282	2.246	2.254	2.177	2.123	1.803	1.953	2.109	2.070	1.807	2.634	2.081	1.631	2.094	2.684	2.075	1.913	1.650
I(C:N)	2.380	2.253	2.181	2.385	2.103	2.283	2.170	2.285	2.294	2.082	2.176	2.559	2.179	2.259	2.386	2.265	2.330	2.157	2.052	2.502	2.104	2.180	2.312	2.360	2.047	2.291	2.460	2.322	2.198	2.333

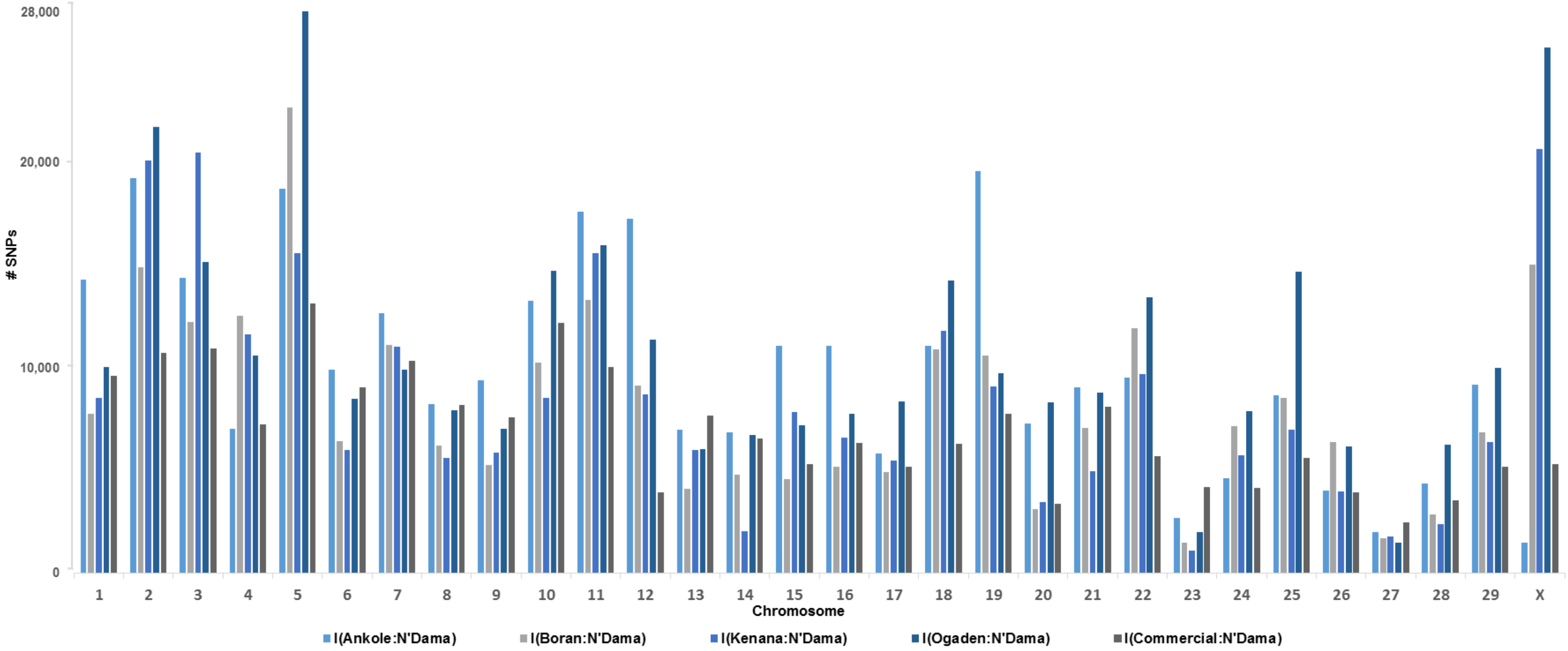

Figure S1. The table and graph below represent the ratio of the extracted SNPs to all the SNPs and the number of extracted SNPs based on MI on each chromosome between N'Dama and five breeds including Ankole, Boran, Kenana, Ogaden and commercial breeds. The ratio is in negative log-scale; thus, red or blue table cells indicate lower or higher ratio values, respectively.

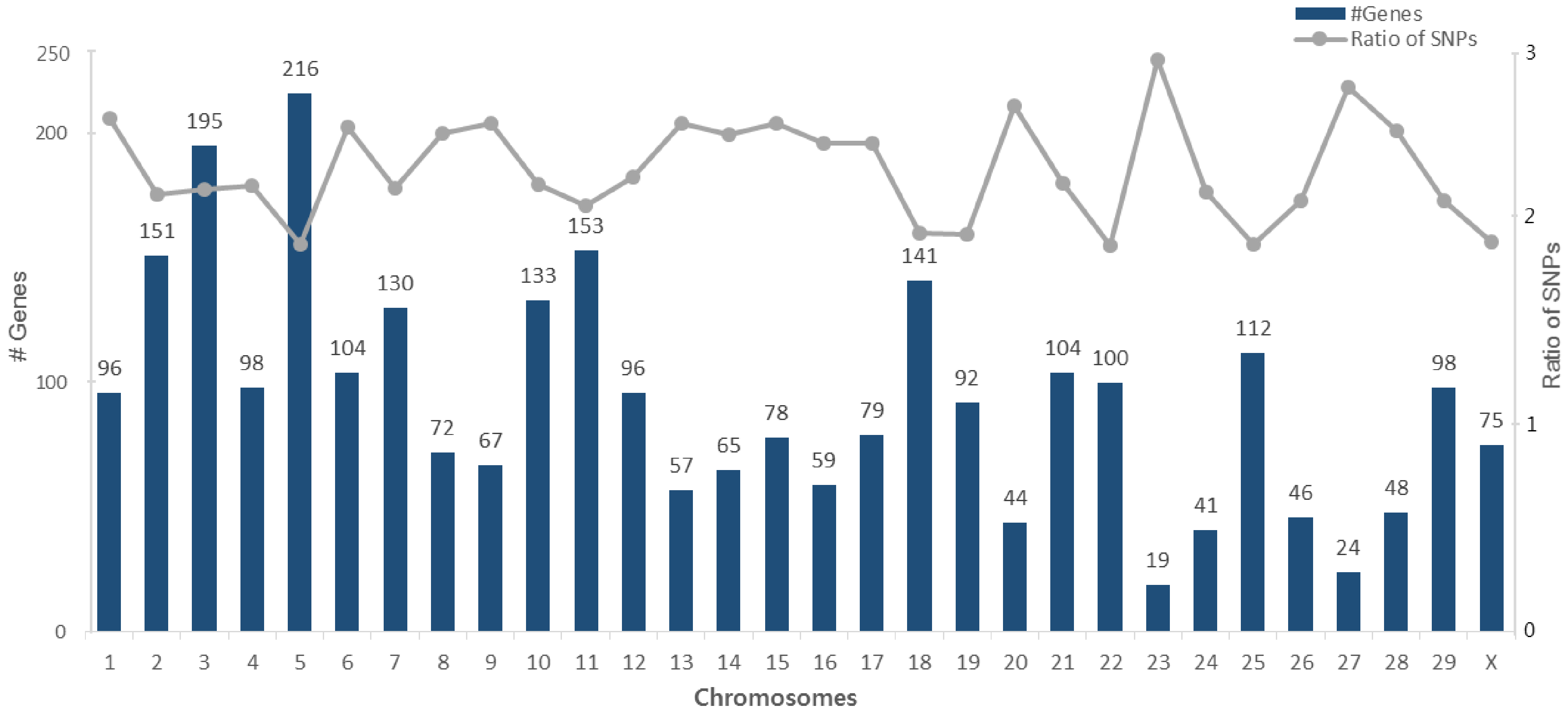

Figure S2. Ratio of the SNPs identified by MI analysis between N'Dama and Ogaden breeds to all the SNPs on each chromosome and below it, the blue bar chart of the number of common genes including the identified SNPs.

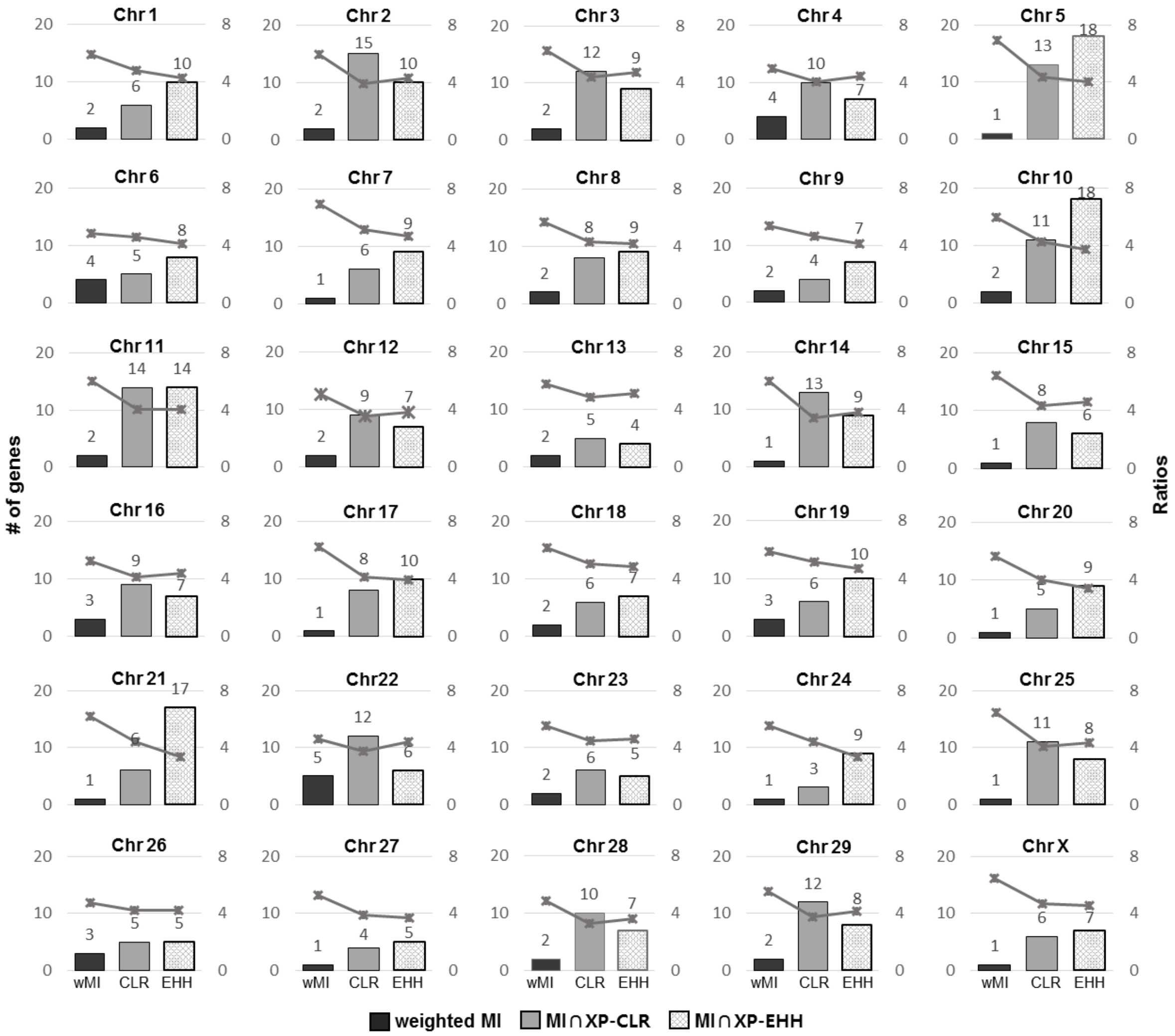

Figure S3. Distribution of the numbers and the log ratios of annotated genes including SNPs distinguishing between N'Dama and Ogaden in each chromosome.

Black, grey and patterned light grey bars indicate the numbers of annotated genes including SNPs identified by wMI, the intersection of MI and XP-CLR, and MI and XP-EHH with a significant *p*-value level (1.0e-2). Line graphs above the bars represent the ratios of the identified genes to the total genes on each chromosome. The value is in negative log scale, thus a lower value denotes a higher ratio. For all graphs, values on the left y axis represent the numbers of genes and a right y axis indicates the ratio.

SNP Position	Ogaden	N'Dama
25:33419222	A/G (5), A/A (3), G/G (1)	A/G (5), G/G (5)
25:33420542	C/T (4), C/C (2), T/T (3)	T/T (10)
25:33423292	A/A (5), A/C (5)	A/A (10)
25:33424142	C/C (4), C/T (3), T/T (2)	T/T (8), C/T (2)
25:33431774	G/G (4), A/G (3), A/A (2)	A/A (8), A/G (2)
25:33435266	C/C (4), C/T (3), T/T (2)	T/T (8), C/T (2)
25:33449887	T/T (4), C/T (4), C/C (1)	C/T (5), T/T (5),
25:33450629	C/T (6), C/C (3)	C/T (5), T/T (5),
25:33450764	A/G (4), G/G (4), A/A (1)	A/G (5), G/G (5)
25:33450812	C/G (4), G/G (4), C/C (1)	C/G (5), G/G (5)
25:33450816	C/G (4), C/C (4), G/G (1)	C/G (5), C/C (5)
25:33451502	C/C (7), A/C (2)	C/C (6), A/C (4)
25:33457079	A/G (4), A/A (3), G/G (2)	A/A (5), A/G (5)
25:33460436	C/C (4), C/G (4), G/G (1)	C/G (5), G/G (5)
25:33467393	A/A (7), A/T (2)	A/A (6), A/T (4)
25:33470163	C/T (4), T/T (3), C/C (2)	C/T (5), T/T (5)
25:33483611	G/G (7), C/G (2)	C/C (5), C/G (5)
25:33484106	G/G (9)	A/A (5), A/G (5)
25:33492065	G/G (6), A/G (3)	A/A (5), A/G (5)
25:33496829	C/C (9)	T/T (5), T/C (5)
25:33497774	C/C (5), C/T (3), T/T (1)	T/T (8), T/C (2)
25:33497776	G/G (5), C/G (3), C/C (1)	C/C (8), C/G (2)
25:33498302	A/G (5), A/A (4)	A/G (5), G/G (5)
25:33499162	G/G (9)	A/A (5), A/G (5)
25:33506430	G/G (9)	A/A (5), A/G (5)
25:33515287	C/C (6), C/T (2), T/T (1)	C/C (10)
25:33517483	G/G (9)	A/A (5), A/G (5)

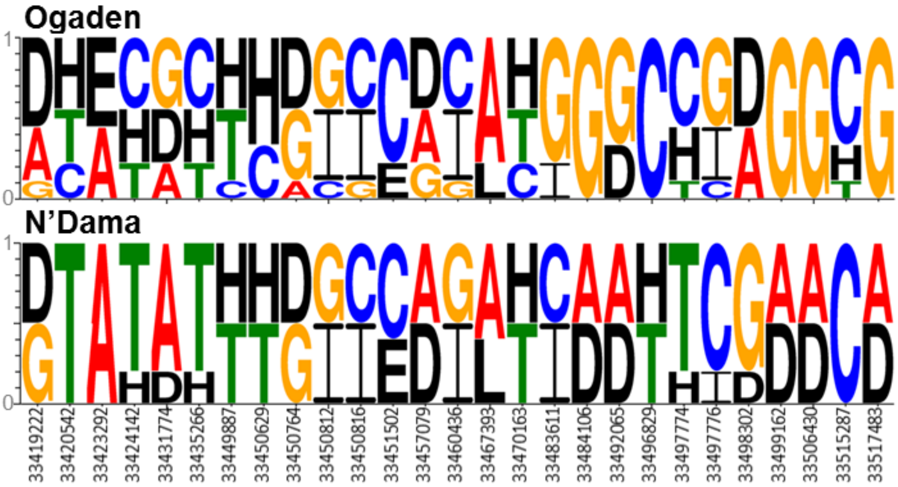

Figure S4. Genotype profiles of GTF2IRD1 between Ogaden and N'Dama breeds

Upper table shows the types of SNP alleles for each breed. Values in the parentheses indicate the numbers of samples with each allele for the gene. A figure reveals the patterns of SNP alleles for detecting each position of GTF2IRD.

SNP Position	Ogaden	N'Dama
17:63406027	C/C (6), C/T (3)	T/T (10)
17:63408262	C/C (6), C/T (3)	T/T (10)
17:63411748	A/A (6), A/G (3)	G/G (10)
17:63420138	A/A (6), A/G (3)	G/G (10)
17:63420223	C/C (5), C/T (4)	T/T (10)
17:63384511	A/G (6), A/A (2), G/G (1)	A/A (10)
17:63391421	C/T (5), T/T (3), C/C (1)	T/T (10)
17:63420322	G/G (6), G/T (3)	T/T (10)
17:63447907	A/G (5), G/G (4)	A/A (10)
17:63449295	A/G (5), A/A (4)	G/G (10)
17:63449830	C/T (5), C/C (4)	T/T (10)
17:63451255	G/T (5), G/G (4)	T/T (10)
17:63418110	A/A (6), A/C (3)	C/C (10)
17:63381362	A/G (6), G/G (3)	G/G (10)
17:63382211	A/C (5), C/C (4)	C/C (10)
17:63410737	G/G (9)	G/G (10)
17:63445573	A/A (7), A/G (2)	A/A (10)
17:63445613	T/T (7), C/T (2)	T/T (10)
17:63446868	A/A (7), A/G (2)	A/A (10)
17:63447430	T/T (7), C/T (2)	T/T (10)
17:63449805	A/A (7), A/G (2)	A/A (10)

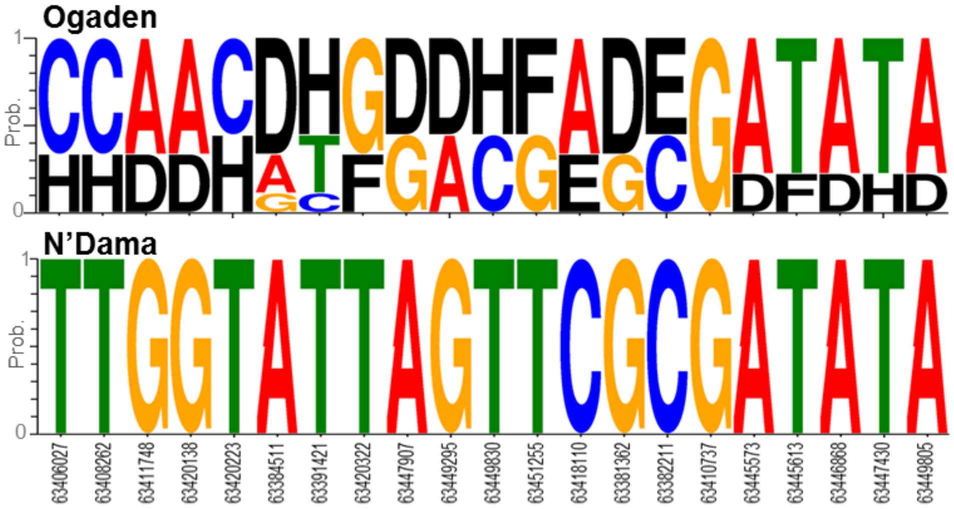

Figure S5. Genotype profiles of RASAL1 between Ogaden and N'Dama breeds

Upper table reveals the types of SNP alleles for each breed. Values in the parentheses indicate the numbers of samples with each allele for the gene. A figure displays the patterns of SNP alleles for detecting each position of RASAL1.

References

1. Eudy, J. D. *et al*. Mutation of a gene encoding a protein with extracellular matrix motifs in Usher syndrome type IIa. *Science*. **280**, 1753-1757 (1998).

2. Nouws, J. *et al*. Acyl-CoA dehydrogenase 9 is required for the biogenesis of oxidative phosphorylation complex I. *Cell Metabolism*. **12**, 283-294 (2010).

3. Nouws, J., Te Brinke, H., Nijtmans, L. G. & Houten, S. M. ACAD9, a complex I assembly factor with a moonlighting function in fatty acid oxidation deficiencies. *Hum Mol Genet.* **23**, 1311-1319 (2014).

4. Díaz-Perales, A. *et al*. Identification and characterization of human archaemetzincin-1 and -2, two novel members of a family of metalloproteases widely distributed in Archaea. *J Biol Chem.* **280**, 30367-30375 (2005).

5. Liu, Q. *et al*. NYD-SP15: a novel gene potentially involved in regulating testicular development and spermatogenesis. *Biochem Genet.* **44**, 409-423 (2006).

6. Eudy, J. D. *et al*. Isolation of a novel human homologue of the gene coding for echinoderm microtubule-associated protein (EMAP) from the Usher syndrome type 1a locus at 14q32. *Genomics.* **43**, 104-106 (1997).

7. Kielar, M. *et al*. Mutations in Eml1 lead to ectopic progenitors and neuronal heterotopia in mouse and human. *Nat Neurosci.* **17**, 923-933 (2014).

8. Arnold, S. J. *et al*. The T-box transcription factor Eomes/Tbr2 regulates neurogenesis in the cortical subventricular zone. *Genes Dev.* **22**, 2479-2484 (2008).

9. Sessa, A. *et al*. Tbr2 directs conversion of radial glia into basal precursors and guides neuronal amplification by indirect neurogenesis in the developing neocortex. *Neuron.* **60**, 56-69 (2008).

10. Endo, Y. *et al*. Eomesodermin controls interleukin-5 production in memory T helper 2 cells through inhibition of activity of the transcription factor GATA3. *Immunity.* **35**, 733-745 (2011).

11. Reed, J. E. *et al*. Expression of cellular adhesion molecule 'OPCML' is down-regulated in gliomas and other brain tumours. *Neuropathol Appl Neurobiol.* **33**, 77-85 (2007).

12. Daimon, M. *et al*. Association of the PIK3C2G gene polymorphisms with type 2 DM in a Japanese population. *Biochem Biophys Res Commun.* **365**, 466-471 (2008).

13. Itoh, A. *et al*. Cloning and expressions of three mammalian homologues of Drosophila slit suggest possible roles for Slit in the formation and maintenance of the nervous system. *Brain Res Mol Brain Res.* **62**, 175-186 (1998).

14. Skutella, T. & Nitsch, R. New molecules for hippocampal development. *Trends Neurosci.* **24**, 107-113 (2001).

15. Bensaad, K. *et al*. TIGAR, a p53-inducible regulator of glycolysis and apoptosis. *Cell.* **126**, 107-120 (2006).

16. Yang, I. V. *et al*. Identification of novel innate immune genes by transcriptional profiling of macrophages stimulated with TLR ligands**.** *Mol Immunol.* **48**, 1886-1895 (2011).

17. Lin, N. *et al*. A novel human dendritic cell-derived C1r-like serine protease analog inhibits complement-mediated cytotoxicity. *Biochem Biophys Res Commun.* **321**, 329-336 (2004).

18. Zhan, R. *et al*. A DEAD-box RNA helicase Ddx54 protein in oligodendrocytes is indispensable for myelination in the central nervous system. *J Neurosci Res.* **91**, 335-348 (2013).

19. Fulton, D. J. Nox5 and the regulation of cellular function. *Antioxid Redox Signal.* **11**, 2443-2452 (2009).

20. Bernard, O. A. *et al.* A new recurrent and specific cryptic translocation, t(5;14)(q35;q32), is associated with expression of the Hox11L2 gene in T acute lymphoblastic leukemia. *Leukemia.* **15**, 1495-1504 (2001).

21. Lee, J. H., Zhou, S. & Smas, C. M. Identification of RANBP16 and RANBP17 as novel interaction partners for the bHLH transcription factor E12. *J Cell Biochem.* **111**, 195-206 (2010).

22. Azzedine, H. *et al.* Mutations in MTMR13, a new pseudophosphatase homologue of MTMR2 and Sbf1, in two families with an autosomal recessive demyelinating form of Charcot-Marie-Tooth disease associated with early-onset glaucoma. *Am J Hum Genet.* **72**, 1141-1153 (2003).

23. Robinson, F. L., Niesman, I. R., Beiswenger, K. K. & Dixon, J. E. Loss of the inactive myotubularin-related phosphatase Mtmr13 leads to a Charcot-Marie-Tooth 4B2-like peripheral neuropathy in mice. *Proc Natl Acad Sci USA.* **105**, 4916-4921 (2008).

24. Ng, A. A., Logan, A. M., Schmidt, E. J. & Robinson, F. L. The CMT4B disease-causing phosphatases Mtmr2 and Mtmr13 localize to the Schwann cell cytoplasm and endomembrane compartments, where they depend upon each other to achieve wild-type levels of protein expression. *Hum Molec Genet*. **22**, 1493-1506 (2013).
